# Experimental and Modeling-Based Approaches for Mechanistic Understanding of Pan Coating Process—A Detailed Review

**DOI:** 10.3390/pharmaceutics18010019

**Published:** 2025-12-23

**Authors:** Behrad Aminahmadi, Elise Vaes, Filip Willemse, Domenica Braile, Luz Naranjo Gomez, Sune Klint Andersen, Thomas De Beer, Ashish Kumar

**Affiliations:** 1 Pharmaceutical Engineering Research Group (PharmaEng), Department of Pharmaceutical Analysis, Ghent University, Campus Heymans, Ottergemsesteenweg 460, 9000 Gent, Oost-Vlaanderen, Belgium; behrad.aminahmadi@ugent.be; 2 Laboratory of Pharmaceutical Process Analytical Technology (LPPAT), Department of Pharmaceutical Analysis, Ghent University, Campus Heymans, Ottergemsesteenweg 460, 9000 Gent, Oost-Vlaanderen, Belgium; 3 Janssen Pharmaceutica NV, a Johnson & Johnson Company, Turnhoutseweg 30, 2340 Antwerp, Beerse, Belgiumsander83@its.jnj.com (S.K.A.)

**Keywords:** tablet film coating, pharmaceutical process modelling, thermodynamic modelling, spray dynamics, discrete element method, process analytical technology, critical quality attributes, data-driven modelling, scale-up

## Abstract

Tablet film coating is governed by three interrelated phenomena, namely, tablet mixing, coating-liquid spraying, and liquid evaporation, which dominate the critical quality attributes (CQAs) of the final product. This review examines how differences in coater design, key process parameters, and quality control strategies impact these phenomena and ultimately affect inter-tablet and intra-tablet coating variability. Two complementary approaches for understanding and optimizing the process are evaluated. The experimental approach, involving Design of Experiments (DoE), retrospective data analysis, and advanced Process Analytical Technology (PAT), provides empirical insight into factor–response relationships and enables real-time quality assurance. Simultaneously, model-based approaches, including thermodynamic, spray-dynamics, and particle-dynamics modelling, offer mechanistic understanding of heat and mass transfer, droplet deposition patterns, and tablet motion. Although these sub-models have advanced considerably over the years, a predictive model that treats the coating process in its entirety is still missing. Overall, this review underscores that future advancements will require integrating experimental and model-based methodologies to achieve robust, quality-driven, and predictive control of tablet film coating processes.

## 1. Introduction

Film coating is a key process step in pharmaceutical manufacturing, involving the application of a thin polymer layer onto solid dosage forms such as tablets, capsules, or beads. This review examines film coating processes using aqueous or organic solvent formulations applied in conventional drum coaters. Furthermore, the purpose of this review is to critically discuss the mechanistic understanding of the tablet film coating process. The various applications of film coating have been comprehensively reviewed by Seo et al. [1]; readers interested in specific application domains are referred to that work.

Film coating process can enhance product stability, improve bioavailability, and facilitate swallowing, ultimately contributing to better patient compliance. Additionally, film coating enhances the aesthetic appeal of pharmaceutical products and enables the development of modified-release formulations through the use of functional coatings. In pan coating, tablet cores are loaded into a rotating drum, where the motion of the drum facilitates both radial and axial mixing. In essence, the coating process consists of repeated exposure of solid dosage forms to a sprayed solution containing a polymer dissolved or dispersed in a solvent. As the drum rotates, gravitational force leads to the downward cascading of the tablets, consequently exposing a new layer to the spray zone. As a particle passes through the spray zone, it receives a partial coating, with the distribution and thickness influenced by local process conditions. Subsequently, the coated particle moves into a drying region where solvent evaporation, typically facilitated by heated air, leads to the solidification of the film. While the cycle of spraying and drying is repeated to achieve the desired coating thickness and uniformity [2], this alone does not guarantee the desired thickness or uniformity. Ensuring stable process parameters, especially consistent movement of tablets through the spray zone, is essential in the pan coating process. Radial mixing occurs more rapidly, ensuring uniform exposure of tablets to the spray zone, while axial mixing is comparatively slower. To enhance axial movement and improve overall mixing efficiency, baffles are often incorporated within the drum [3]. This is because the passage frequency and residence time of tablets in the spray zone determine coating uniformity at both the batch and individual tablet levels. However, some tablets may bypass the spray as they become trapped in stagnant or slow-moving regions of the tablet bed [2].

## 2. Tablet Coating Equipment and Process Operation Modes

### 2.1. Different Types of Coaters and Their Specifications

Drum coaters are available in various designs, which can be broadly classified as fully perforated, semi-perforated, and non-perforated drums. These classifications influence the design of the drying airflow and air handling systems. In addition to perforation type, coaters differ in their internal baffle arrangements and airflow configurations. Most conventional drum coaters, often referred to as cascading systems, operate based on the cascading motion of tablets from the top of the bed, with some particles passing through the spray zone. This spray zone is typically created by one or more nozzles positioned above the tablet bed, directing the coating solution downward [4]. However, the GEA ConsiGma™ semi-continuous coater [5] deviates significantly from this conventional mode of operation. In this system, tablets adhere to the drum wall due to high rotation speeds, are deflected into flight by air knives, coated via a spray system located beneath their trajectory, and then descend under gravity to rejoin the bed on the opposite side of the drum [6,7]. Despite such novel designs, the scope of this review is limited to conventional cascading drum coaters, which remain the standard in industrial practice.

Cascading drum coaters are generally categorized into (bi)conical and cylindrical types based on their structural design. For cylindrical coaters, L.B. Bohle [8] offers pans with patented helical baffles, while IMA Thomas Processing includes cylindrical designs in its FLEX series [9]. Other suppliers, such as IMA, IMA Thomas, Glatt, Syntegon/Bosch/Manesty, O’Hara, Freund, and Loedige, primarily provide biconical pans, each featuring their own baffle designs and nozzle setups.

The primary distinction between (bi)conical and cylindrical coating drums lies in their spray surface area. Cylindrical coating pans have a greater length-to-diameter ratio, resulting in a larger spray surface than conical pans. Additionally, cylindrical drums feature shallower bed depths, which enhance mixing efficiency by allowing better tablet movement and more uniform exposure to the spray zone. However, it is important to note that mixing efficiency is also significantly influenced by the design and positioning of baffles. The height of the baffles plays a crucial role in determining the thickness of the tablet cascade flowing over them, which in turn influences the velocity at which the tablets fall [10]. Thus, the enhanced mixing observed in cylindrical drums is often a combined result of both the shallow bed geometry and optimized baffle configurations.

**Figure 1:**
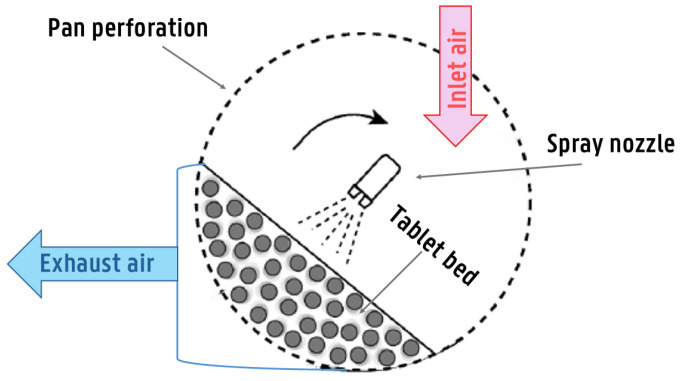
Schematic view of a typical conical drum.

**Figure 2:**
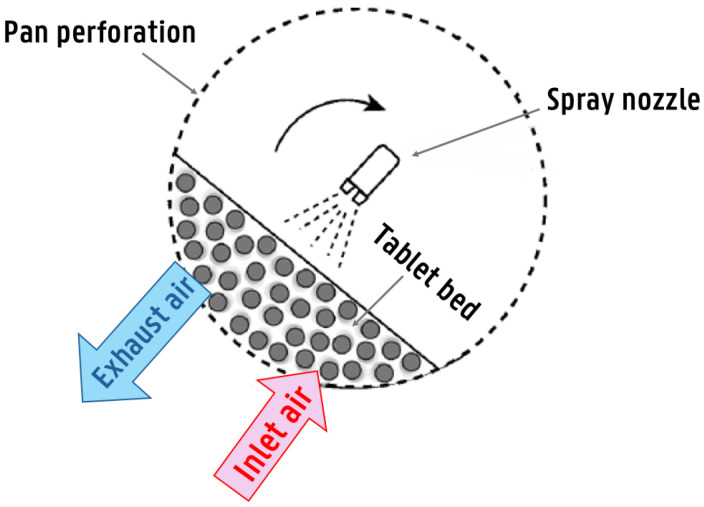
Schematic view of Bohle coating systems.

Side-vented or (bi)conical drums have a co-current air flow and spraying direction (Figure 1). Hence, spray droplets are exposed to the hottest and unsaturated air when traveling from the spray gun to the tablet bed, making coating with these devices more prone to spray drying. For non-perforated drums, air is introduced via air baffles. On the other hand, both the Bohle coater and IMA cylindrical drums feature cylindrical, perforated drums, where hot air is introduced through an air duct located below the tablet bed (Figure 2). The main advantage of this air handling approach is improved drying efficiency. However, a disadvantage is the increased disruption of spray droplet deposition due to the counter-current airflow and spraying direction. It is worth mentioning that conventional biconical pans are more commonly used in the industry than cylindrical ones. However, coaters with fully perforated drums and advanced mixing baffles have recently gained significant attention, especially in active coatings.

### 2.2. Coating Processes

Most film coating processes are traditionally executed in batch mode. However, there has been a growing demand for a transition towards continuous manufacturing in recent times. This transition is motivated by a need for improved efficiency, cost savings achieved through reduced labor and handling, and consistent quality with continuous processing. In this subsection, different operational modes available for the pan coating processes are introduced.

#### 2.2.1. Batch Mode

In this process, a batch of tablets is placed into a drum, followed by the spraying of the coating material onto the surface of the tablets. Additionally, hot air is introduced into the pan to initiate evaporative drying and forms a thin film of coating suspension on the tablets’ surface. Once the desired weight gain is achieved, the coated tablets are removed from the pan. Subsequently, the pan is cleaned, and a new batch of tablets can be loaded for the next coating cycle [11]. Batch coating performance is governed by several process parameters, including pan rotation speed, spray rate, atomization air pressure, gun-to-bed distance, inlet air temperature and humidity, and coating formulation properties such as solid content and viscosity. These parameters collectively impact droplet deposition, drying rate, and the residence time of tablets within the spray zone. Achieving an optimal balance between wetting and drying is critical to avoid defects such as overwetting, spray drying, or rough surfaces. Additionally, batch size is defined by pan load which requires scale up consideration during process transfer.

#### 2.2.2. Continuous Mode

Several manufacturers, such as GEA’s ConsiGma^R^ [5], O’Hara’s Fastcoat^TM^ series [12], IMA’s CROMA [13], and Thomas Processing’s Flex CTC and Accela-CTC 500 [9], offer continuous solutions for film coating. The general principle of continuous coating is the same as that of batch processing. Continuous film-coating processes utilize an elongated side-vented coating pan. Uncoated tablets are continuously fed into the rotating pan at one end, passing under a series of spray guns, and removed fully coated at the opposite end. The typical residence time for this process is approximately 15 min [14,15]. Continuous coating processes have some advantages, such as:

- High throughput rate (up to 1000 to 2000 kg/h) [14];
- Reduced product exposure to severe process conditions (heat, moisture, and mechanical stress) due to shorter processing time [14];
- Enabling implementation of real-time sensor and in-line quality control measurements [15];
- Tablets spend more time on the surface due to the shallower tablet bed [15].

To maintain these advantages, important attention should be paid to tablet mixing in continuous coaters, as if back-mixing occurs, tablets will move backward, which causes tablets to have different residence times inside the drum and raises quality concerns. To ensure tablets’ forward movement, baffles should be designed in a way that facilitates inlet-to-outlet movement of tablets [15,16,17].

For many years, continuous film coating systems could not gain sufficient acceptance. A primary limitation was that tablets processed during start-up and shut-down did not receive uniform coating, often resulting in discarding those tablets, which meant product loss and reduced efficiency [18]. More recently, however, advanced continuous coater designs have been developed to specifically address these challenges. O’Hara Technologies, for example, introduced the Fastcoat^TM^ series [12] featuring a zero-waste strategy, which ensures that tablets passing through the equipment during start-up and shut-down are coated under controlled conditions and therefore do not need to be discarded [15]. Similarly, continuous coaters such as the Flex CTC and Accela-CTC 500 [9] from Thomas Processing incorporate coordinated gun-sequencing strategies that maintain consistent coating quality from the beginning to the end of the process [15]. Process-level studies also support the finding of improved inter-tablet uniformity with continuous coaters. Suzzi et al. [19] demonstrated that, when operated at appropriate fill levels, continuous coaters exhibit low variance in tablet residence times, contributing to reduced inter-tablet coating variability [19]. Barimani et al. [20] provided further evidence for consistent quality of the coating by analyzing coating quality in large datasets of continuously coated tablets [20]. Additionally, mechanistic modelling by Kemp et al. [6] highlighted that the comparatively thin tablet bed in continuous coaters allows each tablet to enter the spray zone more frequently, receiving smaller and more frequent coating doses. This repeated exposure ultimately promotes a more uniform coating distribution across the tablets [6].

Finally, the remaining question could be the challenge of integrating coating directly after tableting in a continuous line. This challenge arises from the elastic recovery of freshly compressed tablets. Because many excipients undergo gradual post-compression expansion, tablets may increase in size after coating has been applied. This dimensional change can disrupt film adhesion and uniformity, representing a significant risk to product quality [15].

#### 2.2.3. Semi-Continuous

Semi-continuous tablet coating offers a hybrid solution between batch and continuous processing, enhancing throughput while maintaining operational flexibility. Examples of semi-continuous coaters include Driam’s DRIACONTI-T pharma LAB^®^ and L.B. Bohle’s KOCO^®^ coater [15]. These systems consist of a series of adjacent coating chambers, each equipped with its own spray nozzle and separated by flaps that prevent tablet back-mixing. They operate continuously or cyclically over multiple batches without requiring a full shutdown between runs. Tablets are loaded, pre-heated, and coated sequentially as they move from one chamber to the next, with the coating process completed in the final chamber, which ejects the finished tablets. This setup enables uniform coating and supports the use of different formulations in each chamber, while avoiding common issues in continuous processing, such as start-up variability and product recirculation. Scale-up is achievable by increasing the size or number of chambers, making these systems well-suited for mid-scale production environments [15,21].

This article is organized as follows: Section 2 discusses the key process parameters and their tablet film coating process, Section 3 explores modeling techniques, Section 4 discusses spray atomization in the context of the tablet coating process, Section 5 presents scale-up approaches, Section 6 covers process analytical technologies, and Section 7 concludes with future perspectives.

## 3. Key Process Parameters and Their Impact on the Coating Quality

A comprehensive understanding of the process, including the roles of individual components and the interactions among critical variables, is essential to ensuring product quality and performance. Therefore, in this section, key process parameters are introduced, and their impact on the final coating quality is discussed.

In coating processes involving perforated drums, the parameters influencing performance and product quality can be broadly categorized into pan- and tablet-related parameters, the thermodynamic condition of the air, and spray-related parameters, which are commonly referred to as mechanical, thermodynamic and spray dynamics factors [4].

Pan- and tablet-related parameters include the drum diameter and depth, pan speed, tablet bed load, tablet size, shape, and mass, the design of mixing baffles, the number and type of spray nozzles, and the properties of tablets, such as size, shape, bulk density, hardness, surface roughness, and hydrophobicity. These factors collectively determine mixing efficiency, residence time distribution within the spray zone, and the overall exposure of tablet surfaces to the sprayed coating. Importantly, the thermodynamic conditions of the air, namely inlet and exhaust air flow rates, inlet air temperature, and inlet air humidity or dew point, govern both heat and mass transfer for solvent evaporation, and therefore directly influence drying capacity, process stability, and the risk of coating defects. Furthermore, spray-related parameters encompass the overall and per-nozzle spray rate, airflow conditions (including inlet and exhaust air flow, temperature, and humidity), atomizing and pattern air pressure, dispersion characteristics, the distance between spray nozzles and the tablet bed, the distance between nozzles, and the total coating time [22].

**Figure 3:**
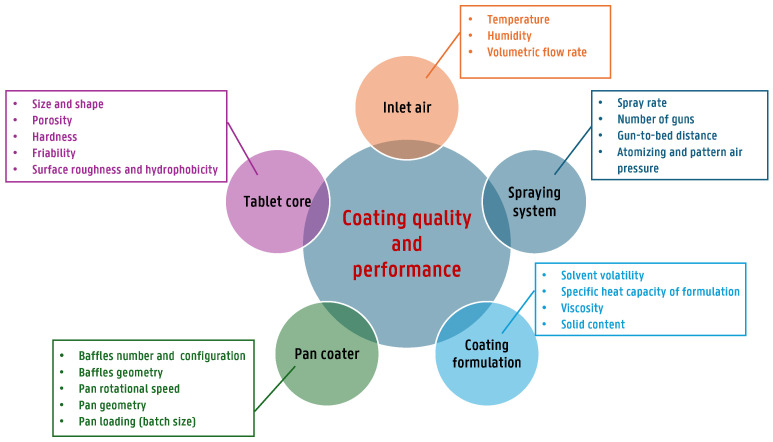
Schematic overview of process-related and material-related variables affecting tablet-coating quality and performance, presented in a radial model to highlight their interconnected roles.

Understanding and optimizing these parameters (further discussed in detail in Section 3.1), as illustrated in Figure 3, are essential for achieving uniform coatings and ensuring the quality of the final product. Depending on how these process parameters are controlled, undesired effects such as picking and sticking, logo bridging or filling, orange peel effects, and erosion may occur due to overwetting, while cracking, peeling, and color variation can result from excessive drying [23,24].

Polymer film uniformity is one of the most important quality attributes for functional coatings and can be categorized into inter-tablet and intra-tablet coating uniformity [25]. Inter-tablet coating uniformity refers to the coating uniformity among different tablets and is primarily influenced by process parameters such as pan speed, tablet load in the drum, drum size and geometry, as well as mixing dynamics and tablet distribution within the spraying zone. In contrast, intra-tablet coating uniformity refers to the evenness of the coating on the surface of an individual tablet. This is primarily influenced by tablet size, shape, and orientation within the spraying zone. Round tablets exhibit lower intra-tablet variability, while oval tablets tend to show higher intra-tablet variability [26]. Uniformity tends to decrease when tablets adopt a preferred orientation [27]. Flatter tablets, for example, often orient themselves with the flat surface exposed to the spray. It has also been concluded that nearly all spherical tablets exhibit less variability in the thickness of the coating layer [26]. Furthermore, it has been experimentally confirmed that even with spherical shapes, preferred orientations can occur. This highlights the importance of using mixing baffles, which facilitate the redirection of tablets within the spraying zone [28]. Residence time represents the total accumulated time a tablet spends in the spraying zone over multiple passages throughout the coating process [29]. Therefore, the weight gain, or the amount of coating dispersion that each tablet receives, is proportional to the residence time [30]. Thus, knowing the residence time distribution of tablets in a batch makes it possible to determine their overall coating mass variability.

### 3.1. Coating Critical Factors—A Way to Control Coating Quality

To control the final coating quality, different critical factors need to be examined and optimized. These factors can be classified into four groups: coating formulation factors, air parameters, uniformity of the spray application, and uniformity of tablet movement.

#### 3.1.1. Coating Formulation Factors

A film coating formulation often contains polymers, plasticizers, colorants, and solvents. When selecting a coating formulation, the wetting properties of the tablet substrate should also be considered, as they affect droplet spreading and penetration and can consequently lead to defects such as logo bridging [31]. Furthermore, the application of the coating is also a determining factor in formulation; for example, in functional coatings, a higher solid content (20–30%) is usually used in the coating formulation [32].

A higher solid content in the coating formulation is preferred because it reduces the amount of solvent that must be evaporated, thereby shortening the overall coating time. However, shorter coating time reduces the residence time of tablets in the spray zone, thereby increasing the risk of coating non-uniformity [33]. Moreover, excessively high solid content can increase the viscosity of the solution or suspension, leading to processing challenges [31]. Higher viscosity affects the spatial distribution of droplets and increases the number of large droplets that tend to travel toward the edge of the spray cone [34]. A higher solid content can also lead to a rough coating surface if the spray droplet size and drying conditions are not properly adjusted. Considering that changes in solid content and formulation viscosity can influence spray droplet characteristics, it is important to fine-tune the spray parameters accordingly [31].

#### 3.1.2. Air Parameters Determining Evaporative Performance

The film coating process is considered an adiabatic evaporative cooling process, meaning that all factors influencing evaporation directly impact the final coating quality. Key parameters include inlet air volume, temperature, and dew point, spray rate, spray gun-to-bed distance, and tablet and exhaust air temperatures.

Inlet air volume plays a crucial role in evaporation, as it drives solvent removal from the coating film. To maximize coating efficiency, the highest possible inlet air volume should be used without inducing turbulent airflow. Similarly, the dew point temperature reflects the moisture content in the process airstream and preferably must be carefully controlled. Additionally, it is possible to utilize a psychrometric chart, where temperature, dry-bulb temperature (°C), and humidity can be correlated and a coating operating line can be obtained. This can be beneficial for the physical description of the process and may potentially be used in a Quality by Design approach to defining a coating design space [35].

Fluctuations in inlet air volume or dew point can reduce the drying capacity of the system, slowing down solvent evaporation. As a result, the coating process may require a longer duration to achieve the desired endpoint, leading to extended processing times. In addition, such fluctuations can also lead to coating defects, such as spray drying (a dry defect) or sticking and erosion (wet defects), ultimately compromising film quality [23].

#### 3.1.3. Uniformity of the Spray Application

Droplet size and velocity are key parameters directly influenced by spray characteristics such as spray rate, as well as atomization and pattern air pressures [34,36]. Larger droplets are harder to evaporate, increasing the risk of overwetting the tablet bed [37]. Conversely, excessively small droplets may undergo spray drying, where the liquid evaporates before reaching the tablet surface and fail to form a coherent wet film on the tablet surface [38]. Since all atomized sprays produce a distribution of droplet sizes, the gun-to-bed distance must be carefully optimized as a compromise: if too short, larger droplets may not evaporate sufficiently before deposition, while if too long, smaller droplets are more likely to dry completely in flight [23].

Another crucial aspect of spray characteristics is the spray pattern, which determines the number of tablets coated in each pass through the spray zone. The spray system must be configured to span the entire pan width to ensure full coverage of the tablet bed and to minimize the likelihood of unsprayed regions or particle bypass [39]. Pandeya et al. [40] compared circular and elliptical spray shapes and concluded that the spray pattern does not have a major impact on coating variability as long as the spray area is kept the same between different spray patterns. In fact, coating variability generally decreases as the total spray area increases [40]. Porter further reported that a circular pattern leads to a smoother but with a higher risk of localized over-wetted coating compared to the elliptical pattern [41]. Therefore, to achieve a uniform coating, an elliptical spray pattern with a consistent droplet size distribution is ideal.

In coating pans with greater length, multiple spray guns should be used with an appropriate gun-to-gun distance to ensure full tablet bed coverage while minimizing pattern overlap or excessive gaps between spray zones [31].

#### 3.1.4. Uniformity of Tablet Movement

Tablet movement uniformity in the coating pan is an important parameter to be controlled. Uneven movement of tablets leads to uneven coating deposition, which results in variability in coating thickness. Pan speed, tablet size and shape, baffle design, and the number of baffles affect tablet flow, mixing efficiency, and consequently, coating quality.

- Pan speed:
  An optimal pan speed is essential to ensure uniform tablet movement, which in turn results in a uniform coating distribution. In general, the highest pan speed that does not cause defects such as tablet breakage or sticking should be used to enhance mixing and reduce coating variability. It is important to continuously monitor and adjust tablet movement throughout the coating process, as the application of the coating alters the tablet surface, increasing the degree of slip of the tablets and potentially affecting movement dynamics [23].
- Baffle design and number:
  Mixing baffles are primarily designed to enhance both axial and radial mixing by guiding tablet movement between the front and back sections of the coating pan and promoting tumbling throughout the bed. As baffles pass through the tablet bed, they temporarily lift portions of the tablets, creating a wave-like surface across the bed. Depending on the position of the spray gun relative to the peaks and troughs of the lifted tablet surface, the gun-to-bed distance can either increase or decrease. Maintaining minimal fluctuations in this gun-to-bed distance is crucial to ensure that the spray consistently travels the same path, allowing spray droplets to reach the tablets with uniform moisture levels [23]. Chen et al. [42] investigated the impact of baffle shapes on tablet movement dynamics and, consequently, coating uniformity. To achieve this, they compared three different cases: without baffles, Xiaolun^™^ baffles, and a self-designed baffle that is flatter and shorter. They demonstrated that the cases with baffles present better coating uniformity compared to the case without baffles. Comparing the two different baffle shapes revealed that the self-designed baffle provides more uniform coating. This is explained by the higher tablet velocity, which promotes sufficiently frequent and more uniform passage through the spray zone, and therefore leads to more uniform coating [42].

#### 3.1.5. Tablet Shape

Tablet shape plays a critical role in how tablets move and reorient within a rotating pan coater, impact defect rates, and affect the overall coating process. Ketterhagen [27] introduced an Orientation Index (OI) to quantify orientation tendencies and showed that OI increases with aspect ratio; elongated tablets display stronger orientation bias than spherical designs. Tablets with high sphericity or aspect ratios close to unity (e.g., spheres, standard round biconvex tablets) tumble readily and tend not to adopt a preferred orientation in the spray zone and therefore receive relatively uniform coating. In contrast, tablets with low sphericity or high aspect ratios (e.g., almond- or shield-shaped) reorient less frequently and often slide through the spray zone with a preferred face exposed, leading to pronounced intra-tablet coating variability [27]. Wilson et al. [26] experimentally found that coating uniformity decreased in the order: round > oval > capsule > large oval. Greater deviation from a spherical geometry produced increasingly uneven coating, with certain regions receiving consistently thinner film layers [26]. Visualizations of film thickness by Freireich et al. [43] revealed that uniformly coated surfaces for nearly round tablets, whereas oval- and capsule-shaped tablets exhibited thinner regions at highly curved ends and thicker coatings on broad faces [43].

In summary, small, round tablets are generally easier to coat compared to oblong- or oval-shaped tablets and capsules [23]. Irregularly shaped cores often require a higher coating application to sufficiently cover edges and ensure uniformity [26].

Despite these effects on intra-tablet variability, tablet shape has been shown to have minimal influence on mixing behavior within the pan and, therefore, little impact on inter-tablet coating uniformity [27].

### 3.2. Using Retrospective Data to Select Critical Process Parameters

Galí et al. [44] conducted a retrospective analysis of data from 36 commercial coating batches processed in a Bohle BFC40 coating machine. A multivariable analysis of all recorded process parameters was performed to identify potential interdependencies and determine the most critical process parameters (CPPs). Based on the findings, a design of experiments (DOE) was established using the retrospective data.

For each batch, a representative sample of 500 tablets was collected to assess and quantify the process outcomes. The tablets were visually inspected to identify appearance defects such as erosion, white spots, and poor coating uniformity, which were quantified based on the sample size. Their observations highlighted a lack of process robustness, as identical coating parameters sometimes resulted in significant variations in batch quality and appearance.

In the DOE analysis, parameters such as dispersion amount, coating time, cooling time, product temperature, warm-up time, and inlet temperature were considered independent factors. Meanwhile, pan speed, atomizing air, and spray rate were classified as interdependent factors. The study focused on three main responses: erosion, white spots, and coating uniformity.

The analysis was divided into three groups. In the first study, all independent factors were varied alongside pan speed, while atomizing air and spray rate were kept constant. The second study involved varying all independent parameters with atomizing air, keeping pan speed and spray rate constant. Lastly, in the third study, the independent parameters and spray rate were varied, with pan speed and atomizing air maintained at fixed levels.

Applying a higher total volume of coating suspension and prolonged coating times enhance homogeneity. However, spray rate and process duration have an opposing effect, necessitating a lower spray rate to prolong the process. Extending the cooling phase reduces white spot defects and enhances coating uniformity. This may be due to a more gradual temperature reduction across the tablet surface, which minimizes condensation risk and allows the film to stabilize without abrupt thermal stress. Meanwhile, product and inlet temperatures must be carefully regulated to ensure proper polymer film formation. Warm-up times should be minimized but must still allow tablet cores to reach the required temperature. Pre-heating the drum before loading the product may be a viable solution to shorten warm-up time. A lower pan speed is recommended at the beginning of the process to prevent erosion, whereas increasing the pan speed in later stages can enhance coating uniformity. Finally, atomizing air pressure should be maintained at the lower range to optimize coating uniformity and reduce white spots [44]. Excessive atomization air pressure can generate very fine droplets that may evaporate before reaching the tablet bed and undergo the spray-drying phenomenon. This can produce a roughened film surface, and, if droplet spreading, drying kinetics, and gun-to-bed distance are not balanced, cause defects such as logo-filling or an orange-peel appearance [45,46].

### 3.3. Using DOE to Select Critical Process Parameters

A widely adopted industry approach involves investigating the influence of coating formulation and process parameters on coating mass variability through systematically designed experiments combined with statistical analysis and regression modeling [40].

Studies by Porter et al. & Just et al. [33,47] concluded that pan speed, spray rate, inlet air temperature, and the number of spray nozzles all influence inter-tablet coating variability, with greater uniformity achieved at higher pan speeds and with an increased number of spray nozzles. While the studies [33,47] did not specify nozzle number in relation to bed geometry, in practice, the optimal number of spray nozzles should also be considered relative to the tablet bed depth and sprayable area to ensure effective coverage and minimize variability. Additionally, [48] recommended a combination of higher pan speed, longer coating time, lower tablet loading, and reduced atomizing air pressure as optimal conditions for achieving uniform inter-tablet coating. Furthermore, studies by [47,49] suggested that coating uniformity could be improved by lowering the spray rate while increasing the pan speed.

In early experimental development, the boundaries of process parameters should be selected based on operational limits, equipment capabilities, and prior quality-based knowledge, ensuring that the design space reflects both practical feasibility and product performance considerations. While DoE provides valuable insights for optimizing specific formulations or products, it remains limited by these predefined boundaries. To understand how process parameters impact coating performance, and hence those boundaries, more mechanistic understanding of the process is needed. For this reason, computational modeling techniques such as thermodynamic modeling, Section 4.1, discrete element method modeling, Section 4.2, and population balance modeling, Section 4.3, are increasingly utilized to gain a deeper understanding of process dynamics and further enhance optimization efforts. These approaches provide a deeper process understanding and complement DoE by imparting the relation between operational parameters and final product quality. The following sections provide a detailed discussion of these modeling techniques.

## 4. Modeling of the Film Coating Process

### 4.1. Thermodynamic Modeling

In pharmaceutical film coating, evaporative cooling of the coating film serves as the primary mechanism considered in thermodynamic modeling of the process. This process is triggered by thermodynamic parameters (affecting mass and heat transfer between the tablet bed and hot air), coater design, and operating parameters. Thermodynamic factors, including temperature, humidity, and inlet air flow rate, play a vital role in film coating by influencing the drying efficiency and solvent evaporation rate. The selection of inlet air temperature should take into account the dew point of the incoming air to optimize the drying capacity of the process [50]. Thermodynamic models are used to predict key process response parameters, such as tablet and exhaust air temperatures, which influence the final coating quality. In film coating, critical parameters such as exhaust air temperature and humidity reflect the drying conditions within the coating pan. Improper drying can lead to various coating defects; for instance, an overly dry environment may cause surface roughness or blistering, while inadequate drying can result in sticking and/or picking [51]. Therefore, optimizing process parameters based on thermodynamic model predictions is crucial [52,53].

#### 4.1.1. Principles of Thermodynamic Modeling and Model Validation

##### Mass and Energy Balance Equations

The thermodynamic model, based on the First Law of Thermodynamics, is formulated using mass and energy balance equations for a control volume within the tablet bed, which functions as a closed, non-isolated system [54,55]. The mass balance follows the law of conservation of matter. In the energy balance, changes in the internal energy of the system are represented in terms of enthalpy [55]. During evaporation, the coating solution must first be heated from $T_{\mathrm{inlet}\mathrm{coating}}$ to $T_{\mathrm{exhaust}\mathrm{air}}\;$, which is accounted for in the energy balance equation as sensible heat. Additionally, the energy required to evaporate the volatile coating component is included in the energy balance equation as latent heat [54,56]. Furthermore, for formulations containing solvents, the model incorporates solvent-specific thermophysical properties such as vapor pressure, concentration, and enthalpy of evaporation, which determine the driving force for mass transfer and influence the overall drying dynamics. For aqueous formulations, the corresponding properties of water are used.

##### Mass Transfer

The solvent surrounding the tablets in the coating is assumed to be in the free-solvent state. In this scenario, the drying process is driven by the evaporation rate (mass transfer) of the thin water film into the gas phase in contact with it [55].

The mass flux between the film and the gas phase, denoted as ${\dot{M}}_{evap}$, is quantitatively described by Equation (1).

(1) $${\dot{M}}_{evap}=-\frac{dm_{s}}{d\tau }=k_{c}\;A_{\mathrm{film}}\;\left(C_{w}-C_{G}\right)$$

The driving force for this mass transfer is determined by the difference in solvent concentration between the interface, $C_{w}$, and the bulk air, $C_{G}$.

These concentrations are defined by Equations (2) and (3), with $M_{S}$ indicating the molar mass of the solvent [57].

(2) $$C_{w}=\frac{M_{S}\;\phi_{S}^{\text{surf}}\;P_{s}^{\mathrm{sat}}\left(@T_{w}\right)}{R\;T_{w}}$$

(3) $$C_{G}=\frac{M_{S}\;\mathrm{RH}\;P_{s}^{\mathrm{sat}}\left(@T_{G}\right)}{R\;T_{G}}$$

The saturated vapor pressures of the solvent at the film interface, $P_{s}^{\mathrm{sat}}$(@$T_{w}$), and in the drying air, $P_{s}^{\mathrm{sat}}$(@$T_{G}$), are computed using the Antoine equation applicable to pure solvents. The molar gas constant is represented as *R*, while the relative humidity of the drying air is denoted by RH. The formulation of the Antoine equation used to calculate the saturated vapor pressure of water is presented in Equation (4) [58]. One important consideration is to ensure that the water vapor pressure in the air does not exceed the saturation vapor pressure at the tablet surface. To guarantee this, critical and limit moisture levels to regulate the evaporation rate is incorporated [56].

(4) $$P_{s}^{\mathrm{sat}}\left(T\right)=10^{3}\;\exp\left(16.2886-\frac{3816.44}{T-46.13}\right)$$

##### Heat Transfer

Heated air facilitates the evaporation of the solvent of the coating solution on the coated tablet. Once the solvent is removed, a film develops on the surface [59]. The gas passing through the tablet bed is modeled as a single-segment plug flow. Heat transfer between the gas phase and the tablet bed occurs through convection, driven by the temperature gradient between $T_{G}$ and $T_{\mathrm{tb}}$ [55,56]. Equation (5) provides a general description of heat transfer, where *A* represents the surface area in $m^{2}$ over which heat transfer occurs, remaining constant throughout the coating process. Additionally, the coefficient $\alpha_{G\to tb}$ can be determined using the Nusselt number.

(5) $${\dot{q}}_{G\to tb}=\alpha_{G\to tb}\;A\;(T_{G}-T_{\text{tb}})$$

##### Heat Loss to the Drum and Environment

Heat loss within the drum accounts for heat dissipation through the pan material, which is an important but often overlooked aspect in thermodynamic modeling of pan coating. Heat loss should be incorporated using general heat transfer calculations described in [60] and depicted in Equations (6) and (7). To incorporate heat loss, heat transfer at three distinct surfaces must be considered: convection from the air to the inner wall of the drum ($\frac{1}{h_{1}}$), conduction through the drum and jacket thickness ($\frac{d}{K}$), and finally, convection from the outer surface of the jacket to the surroundings ($\frac{1}{h_{2}}$) [61].

(6) $$U=\frac{1}{\frac{1}{h_{1}}+\;\frac{d}{K}+\;\frac{1}{h_{2}}}$$

(7) $$Q_{HLF}=U\;A_{drum}\;\left(T_{G}-T_{Surr}\right)$$

Thermodynamic modeling provides a macroscopic view of the process, which is why most existing studies overlook factors such as spray atomization (droplet size distribution), the distance between the spray nozzle and the bed, number of spray nozzles, spray zone coverage, and the airflow from the spray gun nozzle (composed of both the atomization air and pattern air streams). These factors are recognized as affecting coating uniformity, quality, and droplet particle size. Hence, it is important to take these parameters into account when optimizing the coating process [62].

Thermodynamic models require various input parameters to accurately capture the physics of the tablet film coating process. These inputs are broadly categorized in Table 1.

Beyond defining the governing equations and assumptions, ensuring the predictive reliability of thermodynamic models requires proper calibration and validation. Since coating processes operate under varying conditions, the model must be tested across different ranges of process parameters to confirm its applicability. This involves systematic experimental design and validation techniques to assess the model’s accuracy and generalizability. To ensure that the model is applicable across different ranges of process parameters, it should be calibrated and validated using data from various coating processes. A common practice is to establish a Design of Experiments (DOE) based on selected operating parameters [63]. Depending on whether a full or fractional factorial design is chosen, along with the number of studied factors (variables) and responses, the design will determine the suggested number of coating runs, each with a different combination of operating parameter levels. Furthermore, based on various validation methods, such as k-Fold Cross-Validation, Leave-One-Out Cross-Validation (LOO-CV), and Leave-P-Out Cross-Validation (LPO-CV) [64], a certain number of runs are selected for model calibration, while the remaining runs are used for model validation [65].

#### 4.1.2. Overview of Thermodynamic Models and Their Advancements

Table 2 provides an overview of notable published models, highlighting their assumptions, applications, and advancements in thermodynamic modeling of tablet film coating. Early work by am Ende et al. [54] incorporated experimentally obtained Heat Loss Factor (HLF). Building on this, Page et al. [56] introduced zonal division to capture intra-bed variability, while Rodrigues et al. [55] refined the approach by integrating heat loss and simplifying zonal modeling. These advancements laid the foundation for improved accuracy in coating process simulations, as discussed in later sections.

In contrast, Prpich et al. [66] adapted am Ende et al.’s [54] model for scale-up and to establish process parameters in an unqualified coater within a Quality by Design (QbD) framework. Lastly, Strong [35] proposed a theoretical approach based on psychrometric charts, which has received limited attention in the literature.

Therefore, in the following subsections, we focus on the models developed by am Ende et al. [54], Page et al. [56], and Rodrigues et al. [55] to highlight the major transitions in model development.

##### Incorporating Experimental Heat Loss Factor (HLF)

Am Ende et al. [54] developed a thermodynamic model for solvent-based coating in a side-vented biconical coating pan, considering it as a closed, non-isolated system (Figure 1). The model accounts for heat loss due to thermal gradients from the coating pan to the surrounding environment. The model is based on steady-state conditions, assuming no heat, temperature, or mass variations over time. However, the model does not consider humidity, airflow from the spray nozzle, or the sensible heat of the polymer and plasticizer system. The model assumes perfect mixing within the drum, which means that the temperature of the tablet bed $T_{\mathrm{tablet}}$ is equal to the temperature of the exhaust air $T_{\mathrm{exhaust}\mathrm{air}}\;$.

Earlier studies assumed that the outlet air temperature and tablet bed temperature were nearly the same. Later investigations using infrared gun measurements showed that the tablet bed temperature is generally 2–3 °C cooler than the exhaust air temperature, and these temperature differences can significantly fluctuate depending on sensor placement [67]. More recent studies employing PyroButtons [67,68,69] demonstrated that this difference can be as high as 10 °C, depending on process conditions and batch size. Considering these observations, tablet bed temperature was included as a model output alongside exhaust air temperature in the studies of [55]. Despite these findings, the outlet air temperature is still commonly used as an approximation for tablet bed temperature, with a general assumption that it is 1 °C to 5 °C higher [23].

Since direct estimation of HLF is challenging, it is initially obtained experimentally and then treated as a fitting parameter to minimize the residual sum of squared error between the experimental and predicted values for $T_{\mathrm{air},\mathrm{out}}$. This approach enhances model accuracy by incorporating a key heat loss mechanism that would otherwise introduce errors in predicting energy transfer within the system.

To extend the model’s applicability across different side-vented coating pan designs, am Ende et al. [54] experimentally measured HLF for various pan scales, including 1 kg (HCT-30, LDCS-20), 12 kg (HCT-60), 60 kg (Compulab-36), 120 kg (Accela-Cota-48), and 220 kg (HC-130L). By validating HLF across different scales, they ensured that the model remained robust and adaptable to a wide range of pan designs, making it a valuable tool for process scaling and optimization within this specific equipment train, where coater design remains consistent.

HLF can be theoretically calculated using $h_{\mathrm{loss}}$ = K $\Delta T^{0.25}$ according to [70], where K was estimated to be 0.32. This value was determined by am Ende et al. [54] based on the assumption that the dryer surface consists of four vertical plates and one horizontal plate inside a side-vented biconical coating pan. This equation showed good accuracy in predicting the experimentally obtained HLF value [54].

(8) $$T_{G,\mathrm{out}}=\frac{{\dot{m}}_{G,in}\;C_{p,G}\;T_{G,in}+x_{s}\;{\dot{m}}_{\text{coat}}\;C_{p,s}\;T_{\text{coat}}-x_{s}\;{\dot{m}}_{\text{coat}}\;\Delta H_{\mathrm{vap},s}+HLF\;T_{Surr}}{{\dot{m}}_{G,in}\;C_{p,G}+X_{s}\;{\dot{m}}_{\mathrm{coat}}\;C_{p,s}+HLF}$$

The latent heat of vaporization of the solvent is denoted as $\Delta H_{\mathrm{vap},s}$, and for multiple solvent components, the values are combined based on individual component properties.

The model developed by am Ende et al. [54] has been effectively utilized in the study conducted by Prpich et al. [66]. In this work, the model within a Quality by Design (QbD) framework has been applied to optimize process parameters for scaling up the film coating operation from a Glatt GC1250 coater (Glatt GmbH, Binzen, Germany) to a Glatt GC1500 coater. The model played a crucial role in defining an appropriate range of process conditions for the new coater. Additionally, its predictions helped establish key equipment settings, such as upper and lower inlet air temperature limits, to maintain process consistency across different scales [66].

##### Zonal Division for Enhanced Intra-Bed Variability Representation

Page et al. [56] presented a physical model describing aqueous coating inside a fully perforated drum coater (BLC 5, Bohle lab coater) (Figure 2), which consists of a fully perforated cylindrical drum with two inner and three outer mixing baffles. These baffles and the inclination of the axis of rotation dictate the movement of the tablets inside the drum. Hot air enters from the bottom of the drum, passes through the tablet bed, and exits below it. The tablet bed is divided into two zones: a spray zone and a drying zone. The spray zone consists of the tablets in the top layers of the bed, which are coated and partially dried by the hot air coming from the drying zone. The mass of tablets in spray zone is denoted by $M_{tp1}$. On the other hand, the drying zone corresponds to the tablets in the bottom layers of the bed, which are in direct contact with the hot air. The mass of tablets in drying zone is presented as $M_{tp2}$.

In the Bohle coaters, ribbons attached to the drum wall induce horizontal mixing, causing the tablets to constantly move between zones with an exchange rate of $\psi_{j}$. Over a given period, the number of tablets leaving one zone must match the number entering the next.

(9) $${\psi_{j}}_{1}\;M_{tp1}={\psi_{j}}_{2}\;M_{tp2}$$

Theoretical considerations suggest that a portion or the entire volume of tablets between the ribbons may be transferred to another zone. This volume, $V_{P}$, is determined by multiplying the circular area of the inner ribbon covered by tablets, $A_{P}$, with half the drum’s length, *H*.

(10) $$V_{P}=\frac{A_{P}\;H}{2}$$

The exchange rate, $\psi_{j}$, is influenced by several factors, including an exchange rate constant, $c_{\psi_{j}}$, the height of the tablet bed (filling degree), the radius to the outer ribbon, the heights of the outer and inner ribbons, the base height of the inner ribbon, and the pan speed, $n_{s}$.

(11) $${\psi_{j}}_{2}=c_{\psi_{j}}\;\frac{V_{P}\;\rho_{bulk}}{M_{tp1}+M_{tp2}}\;n_{s}$$

The exchange rate constant, $c_{\psi_{j}}$, defines the fraction of volume $V_{P}$ exchanged during a single drum rotation. The exchange of the tablets between zones should be captured in the model by addition of exchange rate to the mass and energy balances. The exchange rate constant, $c_{\psi_{j}}$, is further treated as an optimization parameter. As discussed earlier, determining the critical and limit moisture contents is essential for evaporative mass transfer calculations. These two values can also be considered as optimization parameters in the thermodynamic model. Additionally, if there is no supplementary data, for example from discrete element method simulations (DEM), on the mass of tablets in each zone ($M_{tp1}$ and $M_{tp2}$), these can also be treated as optimization parameters.

##### Balancing Complexity: Incorporating Heat Loss and Lumped Parameter Modeling

While the model developed by Page et al. [56,71] provided a detailed representation of aqueous coating dynamics, its applicability to coaters with different configurations remains unclear. Additionally, the zonal division complexity introduced in the model may not be essential for control system design. Moreover, the lack of explicit validation for predicted humidity data and the omission of heat loss limited its applicability. To address these limitations, Rodrigues et al. [55] proposed a simplified thermodynamic model for aqueous coating, adapted for a 24-inch perforated biconical pan coater (Labcoat IIX, O’Hara Technologies Inc., Richmond Hill, ON, Canada). By removing the zonal division, their model eliminates the need for tablet exchange rate estimation while incorporating heat loss, enhancing its thermodynamic representation and broadening its applicability.

The control volume in the model developed by Rodrigues et al. [55] consists of three interconnected sub-systems: (1) the tablet bed, encompassing both solid and liquid phases where the spray coating process takes place; (2) the drying air, representing the gas phase; and (3) a component that accounts for water evaporation from the coating solution as droplets travel from the spray gun to the tablet bed.

To simplify the analysis of heat and mass transfer during film coating, a lumped parameter approach is adopted. Rather than accounting for differences across individual tablets or specific regions within the bed, this method assumes the entire tablet bed behaves uniformly. For heat transfer, this means the entire tablet bed is assumed to have the same temperature. For mass transfer, it neglects spatial variations in evaporation and moisture content. This approach is particularly useful in thermodynamic modeling, where capturing the dominant transfer mechanisms is more important than representing individual tablet behavior. This fact motivates the incorporation of a lumped parameter for mass (evaporation rate) and heat transfer calculations.

The evaporation of water, ${\dot{M}}_{evap}$, Equation (12), is governed by the gradient between the partial pressure of water vapor at the tablet surface $P_{s}^{\mathrm{sat}}$($T_{\mathrm{tb}}$), which is considered to be saturated, and the partial pressure of water vapor in the surrounding gas phase $P_{s}^{\mathrm{sat}}$($T_{G}$). The above-mentioned pressures can be calculated using Antoine’s equation [58].

(12) $${\dot{M}}_{evap}=U_{L\to G}\;\left(\frac{P_{s}^{\mathrm{sat}}\;\left(T_{\mathrm{tb}}\right)}{R\;T_{\mathrm{tb}}}-\frac{P_{s}^{\mathrm{sat}}\;\left(T_{G}\right)}{R\;T_{G}}\right)$$

The implemented lumped parameter, $U_{L\to G}$, is defined as the product of the evaporative surface area and the mass transfer coefficient. In this work [55], the driving force for heat transfer is considered to be the temperature gradient between the effective gas temperature ($T_{G}^{*}$) and the tablet bed temperature ($T_{\mathrm{tb}}$). This differs from the reference model [56], where the temperature gradient between the exhaust air and the tablet temperature was assumed as the driving force for heat transfer. The effective gas temperature $T_{G}^{*}$ is determined as a function of the outlet gas temperature $T_{G}$, as shown in Equation (13).

(13) $$T_{G}^{*}=\lambda \;T_{G}$$

Similar to mass transfer, the lumped parameter $\alpha_{L,(G\to tb)}$ is incorporated into the heat transfer calculation, as shown in Equation (14), to account for the product of the heat transfer coefficient and surface area.

(14) $${\dot{q}}_{G\to tb}=\alpha_{L,(G\to tb)}\;\left(T_{G}^{*}-T_{\mathrm{tb}}\right)$$

One key aspect of this model [55] is the introduction of an effective drum mass, $m_{D}^{*}$, in the energy balance equation (Equation (15)) for heat loss calculations. This approach accounts for additional heat sinks, such as the drum shaft and equipment walls, enhancing the alignment between simulated and measured drum temperatures. Moreover, the effective drum mass is treated as an optimization parameter [55].

(15) $$\frac{dq_{D}}{dt}=m_{D}^{*}\;C_{p_{D}}\;\frac{dT_{D}}{dt}={\dot{q}}_{G\to D}-{\dot{q}}_{D\to W}$$

For convective heat loss between the gas and the inner wall of the drum, the temperature gradient is defined between the average inlet and outlet gas temperatures and the drum temperature, as shown in Equation (16). Similarly, for convective heat loss between the outer wall of the drum and the surroundings, the temperature gradient is considered between the drum temperature and the surrounding temperature, as also shown in Equation (16) [55].

(16) $${\dot{q}}_{G\to D}=\alpha_{G\to D}\;\left(\frac{T_{G,in}+T_{G,\mathrm{out}}}{2}-T_{D}\right)$$

(17) $${\dot{q}}_{D\to W}=\alpha_{D\to W}\;\left(T_{D}-T_{Surr}\right)$$

### 4.2. Discrete Element Method Modeling

**Figure 4:**
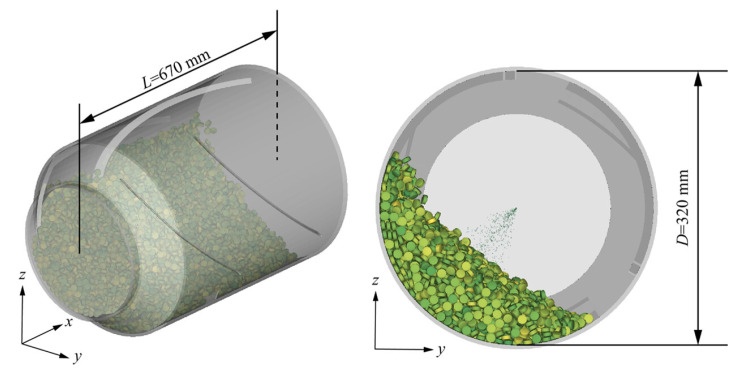
Representative frames from the coating-process simulation within the drum containing baffles. [Reproduced with permission from Liu, Zihan, et al., Industrial & Engineering Chemistry ResearchPharmaceutics; published by American Chemical Society, 2022 [74]].

Understanding tablet movement and interactions within the coating drum is essential for optimizing coating processes, as these dynamics directly impact coating quality, efficiency, and uniformity [72,73]. The DEM provides a powerful tool to simulate tablet motion, which is largely driven by gravitational and inertial forces, along with frequent particle–particle and particle–wall collisions [74,75,76]. In practice, DEM simulations typically handle $10^{5}$–$10^{6}$ tablets, generating fundamental data such as particle positions and velocities. Representative snapshots of the simulated coating process are shown in Figure 4. Additionally, DEM allows tracking of tablets passing through the spray zone at a given time, offering insight into spray distribution and overall coating performance [4,74].

Mellmann [77] illustrates different tablet motion regimes in unbaffled rotating cylinders using a Bed Behavior Diagram, which relates the wall friction coefficient and the Froude number to the filling degree. The aim of this work [77] was to develop simple equations that define the boundaries between different types of bed motion. At very low Froude numbers (below $10^{-4}$), particles mostly slide or surge with limited motion. As the Froude number increases (from around $10^{-4}$ to $10^{-1}$), the motion shifts into rolling and cascading, which are ideal conditions for mixing tablets during coating. These movements keep the particles moving gently and evenly, ensuring a uniform film. If the Froude number increases (between 0.1 and 1), tablets start to cataract, flying along curved paths and hitting each other more forcefully, which can lead to increased impact and potential tablet damage. At values above 1, particles adhere to the drum wall due to strong centrifugal forces, resulting in a centrifuging regime with little to no mixing [77]. The rolling and cascading regimes generally provide the optimal balance between mixing efficiency and minimal tablet damage [78]. DEM has been applied to study these concepts in various studies [42,79,80,81]. These studies have utilized DEM to simulate tablet motion and mixing behavior within the drum, as well as their trajectories and surface flow velocities.

Tablet mixing is also influenced by tablet-specific properties such as shape, size, and density, as well as the characteristics of the coating formulation. Among these, tablet shape has received notable attention in recent years [19]. Using DEM, two common modeling strategies for shape are the multi-sphere (“glued spheres”) method and the polyhedral approach [19,27,74,82,83]. DEM simulations have shown that tablet shape significantly affects intra-tablet coating uniformity, while having minimal influence on inter-tablet variability [27]. Oval- and capsule-shaped tablets tend to have more consistent surface exposure to the spray due to their reduced sphericity, leading to higher intra-tablet variability. On the other hand, more spherical tablets roll more freely and are less likely to expose the same surface to the spray, which in turn decreases intra-tablet variability. While intra-tablet variability tends to stabilize over time, inter-tablet variability declines in inverse proportion to the square root of time [82]. This fundamental difference underscores the importance of considering mixing dynamics, tablet geometry, type and design of coating equipment when aiming for consistent coating performance. Although experiments are needed to validate the DEM model, it remains a promising approach to obtain information that is difficult or impossible to measure directly and to study the impact of different process parameters on the final quality without performing experiments for each case. Depending on the modeling objectives, such as evaluating heat and mass transfer, air–particle interactions, or coating uniformity, airflow dynamics can be incorporated through DEM–CFD coupling [84]. It should be noted that particle cohesion is typically accounted for within the DEM framework through interparticle force models, rather than via CFD coupling [85].

### 4.3. Population Balance Modeling

Population balance modeling (PBM) framework describes the evolution of coating thickness distribution across a population of tablets over time. A calibrated and validated PBM can be used as a quality control tool since it provides insight into inter-tablet uniformity, the coefficient of variation (CV), and the relative standard deviation (RSD) of the coating thickness or weight across tablets [86].

Tablets in the spray zone are sprayed with a certain spray rate and then moved to the drying zone. Each tablet undergoes this cycle multiple times, and during these cycles, tablets are exchanged between the two zones at a steady-state rate. The coating mass distribution depends on the spray and the exchange rates. Tablets have different movement patterns within each zone. Zone 1, where all tablets cascade and are sprayed evenly, can be assumed to have a well-mixed flow pattern. Zone 2, where the tablets move in a direction opposite to the cascading layer and show no relative motion with respect to each other, can be assumed to follow a plug flow pattern. This zone is modeled as a series of *N*-1 ideal mixers connected in sequence [86,87]. The population density of tablets in the spray zone at time *t* with coating mass between *x* and x+dx is represented by $\psi_{1}\left(x,t\right)$. The same definition applies for the drying zone, represented by $\psi_{2}\left(x,t\right)$:

(18) $$\frac{\partial \psi_{i}^{1}}{\partial t}=-\frac{\partial \left(G\psi_{i}^{1}\right)}{\partial x}-\frac{Q_{c}\left(\psi_{s}^{1}-\psi_{e}^{1}\right)}{\beta N_{t}}$$

(19) $$G=\frac{Q_{l}X_{s}}{\beta N_{t}}$$

(20) $$\frac{\partial \psi_{i}^{k}}{\partial t}=-\frac{Q_{c}\left(\psi_{s}^{k}-\psi_{e}^{k}\right)}{\left(\frac{1-\beta }{N-1}\right)N_{t}}$$

In the equations above (Equations (18)–(20)):

β and $Q_{c}$ are parameters that must be obtained from either experiments or DEM simulations before solving the model [40,86]. The terms $\psi_{s}$ and $\psi_{e}$ denote the start (entry) and end (exit) of a region, respectively. The recurrence relation $\psi_{e}^{k}=\psi_{s}^{k-1}$ reflects the fact that the outlet distribution of one region becomes the inlet distribution of the next, consistent with the cyclic motion of tablets in the rotating drum. Some of the important assumptions of this model are:

- There is a constant exchange rate of particles between these regions;
- There is uniform spraying, and the quantity of deposited coating is linked to the duration the particle remains within the spray zone;
- The probability of exchange for a particle with a specific coating amount is linked to the number of particles with the same amount of coating within that region.

#### Compartmental Population Balance Modeling

Compartment models provide a structured way to represent the movement of tablets within the coating pan by dividing it into distinct zones, facilitating the estimation of residence times and transitions between regions. This zonal approach captures the spatial dynamics of tablet circulation during the coating process.

Deposited coating material can be discretized into $N_{c}$ particle mass bins within the range of $N_{c}=\left[w_{i-1};w_{i}\right]$ i = 1, 2, …, $N_{c}$. Each range will have a characteristic coating material amount as $W_{i}=\left[w_{i-1}+w_{i}\right]/2$ [40].

The total number of particles in class $C_{i}$ at time t is denoted by $N_{i}$, while $n_{i}$ denotes the fraction of particles in class $C_{i}$ at the same time [86]. The superscript in Equations (21)–(24) refers to the number assigned to the corresponding perfect mixer [40,86].

(21) $$\frac{N_{i}^{1}}{dt}=G\left(N_{i-1}^{N}-N_{i}^{1}\right)+Q_{c}\left(n_{i}^{N}-n_{i}^{1}\right)$$

(22) $$\frac{N_{i}^{K}}{dt}=Q_{c}\left(n_{i}^{k-1}-n_{i}^{k}\right),\;k=2,\ldots ,N$$

(23) $$n_{i}^{1}=\frac{N_{i}^{1}}{\beta \;N_{t}}$$

(24) $$n_{i}^{k}=\frac{N_{i}^{K}\;\left(N-1\right)}{\left(1-\beta \right)N_{t}}$$

Kumar et al. [87] built the compartment model based on the flow behavior of tablets in a rotating drum rather than on their residence time distributions. The model considered the tablet bed as an outer circulating region and a central region with lower velocity. The circulating loop consists of the spray zone and a part of the bed, which is referred to as the active bed zone. This loop can be assumed as a plug flow. In practice, this can be represented by a series of equally sized continuous stirred-tank reactors (CSTR), which can be an efficient way to capture the residence time distribution. In addition, the center part, also called as the passive bed zone, is assumed to be a single CSTR. Particles are exchanged between the spray zone and the active bed zone at a rate denoted by $N_{loop}$, whereas the exchange rate between the active and passive bed zones is $N_{exch}$. This compartment model follows the principle proposed by Denis et al. [86], with the addition of a non-active (passive bed) zone to the compartment model. For detailed population balance equations, the reader is referred to the original paper [87].

### 4.4. Strengths and Limitations of the Modeling Approaches

Thermodynamic modeling, by integrating energy and mass balance principles, provides insights into key parameters such as exhaust air temperature, tablet bed temperature, and humidity. DEM simulate tablets movement and mixing using Newton’s equations of motion. Detailed information on particle motion, mixing, residence time distribution, and inter- and intra-tablet coating variability can be obtained by employing DEM simulations. The combination of DEM with computational fluid dynamics (CFD) even leverages the accuracy of the prediction by including airflow and spray dynamics [84,88]. PBM, which is computationally more efficient than DEM, can be used to study and understand weight gain evolution among tablets over spraying time. By coupling these modelling approaches, a more comprehensive understanding of the pan coating process can be achieved, leading to improved process control and coating uniformity. Table 3 outlines the key strengths and limitations of the above-mentioned modelling approaches.

## 5. Spray Atomization and Droplet Drying in Transit to the Tablet Bed

**Figure 5:**
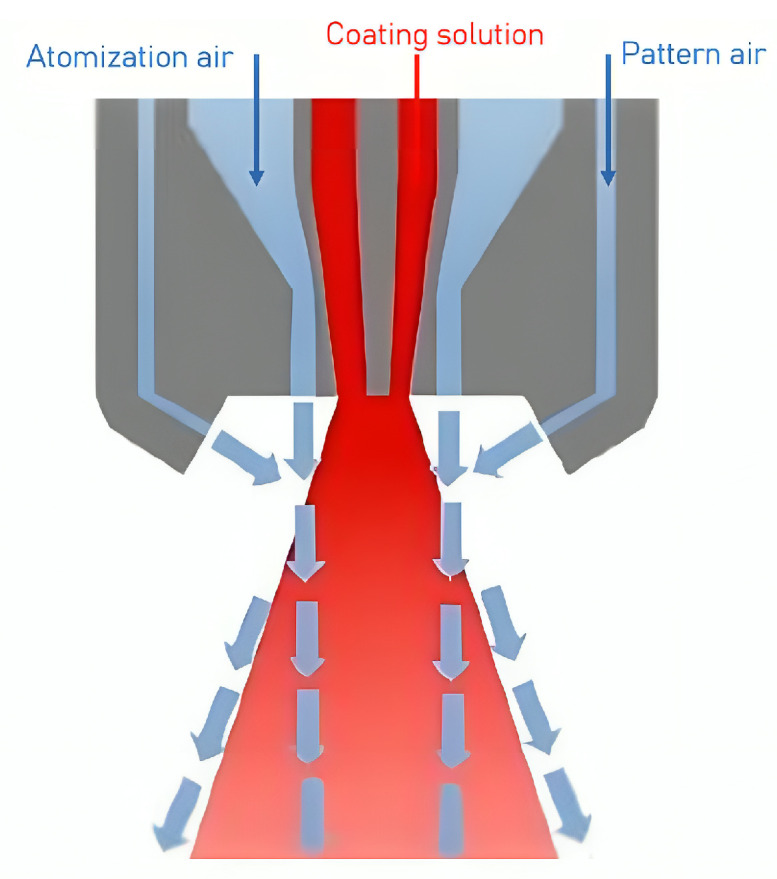
Simplified representation of a spraying system. [Reproduced from Seo, Ki-Soo, et al., Pharmaceutics; published by MDPI, 2020. [1]].

There are two types of spray guns used in tablet coating processes: pneumatic and hydraulic spray guns. Hydraulic guns, due to their wider spray distribution, have limited application in pan coating processes [23]. The pneumatic spray system is the dominant technology and is illustrated in Figure 5. In pneumatic guns, the coating solution is injected through a small orifice at the center of the atomizer, while pressurized air is introduced coaxially through an annular gap at the base of the liquid nozzle. A portion of the pressurized air, 10 % to 20 %, referred to as pattern air in coating processes, flows through auxiliary ports located around the perimeter of the gas cap [23,91]. While the functionality and efficiency of these atomizers have been widely explored [92,93], most research has primarily focused on low-viscosity or Newtonian fluids. Only a few studies have specifically addressed the atomization dynamics of highly viscous or non-Newtonian fluids [91,94].

There are different approaches that are used to account for the spray dynamics in coating processes, including the static spray zone, direct spray droplet modeling, and ray tracing.

- Static spray zone: a geometric region is considered as representative of the spray zone. Particles receive coating based on their residence time within this zone [27,28,30].
- Modeling spray droplets directly: often referred to as the discrete drop method (DDM). This method simulates individual droplet size and velocity. To better approximate spray patterns, droplets are grouped into parcels [74,95].
- Ray tracing: droplet trajectories in this method are represented as virtual rays. A coating event occurs when a ray intersects a particle surface. While this approach is computationally efficient, it is generally less accurate, particularly for low coating masses [83,96].

Each method has strengths and limitations, and they may be used individually or in combination, depending on the desired accuracy and computational feasibility [97]. The following section explores a widely used spray droplet size model in greater detail.

### 5.1. Spray Droplet Size Modeling

A physical mechanism model for spray droplet size describes atomization as a two-stage instability phenomenon. Initially, a primary shear instability develops, leading to the formation of liquid tongues [98,99]. These structures then undergo a secondary Rayleigh–Taylor instability, which further breaks them into droplets [100]. Notably, the diameter of the liquid jet plays a minimal role in this process [93]. Instead, the thickness of the gas boundary layer at the nozzle exit determines the wavelength of the initial instability, influencing how much liquid is exposed to the gas stream [101,102]. In the case of low-viscosity fluids, where viscous forces are negligible, the size of the resulting droplets is primarily governed by surface tension, as it dictates the Rayleigh–Taylor instability wavelength and the breakup of liquid ligaments [93]. The model developed by [91] (Equation (25)) enables the prediction of the Sauter Mean Diameter (SMD) of spray droplets at the spray gun outlet.

(25) $$\begin{array}{cc} \frac{SMD}{D_{l}} & =C_{1}\;\left(1+m_{r}\right)\;{\left(\frac{b_{g}}{D_{l}}\right)}^{1/2}\;\left(\frac{\rho_{l}/\rho_{g}}{Re_{b_{g}}}\right)\\ \; & \;\times \frac{1}{\sqrt{We_{D_{l}}}}\left\{1+C_{2}\;{\left(\frac{D_{l}}{b_{g}}\right)}^{1/6}\;{\left(\frac{Re_{b_{g}}}{\rho_{l}/\rho_{g}}\right)}^{1/12}\;{We_{D_{l}}}^{1/6}\;{Oh}^{2/3}\right\} \end{array}$$

The coefficients $C_{1}$ and $C_{2}$ are empirical constants within the model. $C_{1}$ primarily depends on the gas nozzle geometry, particularly the contraction ratio, as this influences the gas boundary layer thickness at the liquid nozzle exit. $C_{2}$ represents the influence of viscosity on the Rayleigh–Taylor instability in relation to surface tension. It reflects the combined effects of viscosity and surface tension on the instability’s growth rate. The applicability of linear Rayleigh–Taylor instability theory has been validated across a broad parameter range through observations of jet breakup behavior [72].

The SMD estimation relies on the nozzle’s physical characteristics, the rheological properties and the spray rate of the coating liquid, and the volumetric flow rate of the atomizing air [66]. Moreover, turbulence in the liquid stream has a minimal impact on the atomization process. However, a turbulent gas stream can change the atomization dynamics and might necessitate modifying the exponent of $Re_{b_{g}})$ in Equation (25) [72].

The droplet SMD increases significantly as viscosity increases. At lower viscosities, surface tension primarily governs droplet size. However, at higher viscosities, viscous forces dominate the atomization process. Moreover, increased viscosity also extends the distance over which atomization occurs. Both primary and secondary instabilities develop more slowly with higher viscosity. The addition of the Oh and $We_{D_{l}}$ in the model enables it to capture the variation in droplet SMD with increasing viscosity [91].

The previously developed model, Equation (25), lacks consideration of the influence of pattern air on liquid breakup. The impact of pattern air on droplet SMD depends on its interaction with the liquid breakup process. When pattern air jets impinge on the spray after the liquid breakup has begun, their influence on droplet size is minimal since the secondary Rayleigh–Taylor instability already dictates droplet formation. However, if pattern air interacts with the liquid jet while instabilities are still developing, it significantly affects atomization by altering the air velocity distribution. This can be incorporated into the model by averaging the velocities of the atomizing and pattern air streams [72]. Additionally, in setups where pattern air shares the same supply as the atomizing air, it reduces the airflow through the atomizing nozzle, thereby lowering air velocity at the exit of the nozzle. In such cases, the total airflow should be distributed based on nozzle cross-sectional areas and pressure losses to accurately determine air velocity and its impact on atomization [72].

### 5.2. Influence of Operational and Material Parameters on Atomization and Droplet Size

Spray characteristics, such as spray pattern, droplet size distribution, and droplet velocity, play a crucial role in determining spray quality [34]. Furthermore, the position of the spray relative to the tablet bed also influences the spray quality, as it affects spray pattern coverage and intensity across different areas of the bed.

The atomization model described in Section 5.1 depends on several input parameters, which can be classified as operating and material parameters. These include the atomization air flow rate (AA), coating fluid spray rate (SR), pattern air flow rate (PA), viscosity, surface tension, and density.

Coating solutions with high solids have higher viscosity and surface tension, which increase the energy barrier for atomization, resulting in the generation of larger droplets. A global sensitivity analysis (GSA) by [72] reveals that AA and coating fluid viscosity are the two dominant parameters affecting atomization and, consequently, the mean droplet size. The first-order index for AA is significantly higher than that for coating fluid viscosity when considering low-solid-content coating solutions. However, for high-solid-content coating solutions with higher viscosity, the first-order index for coating fluid viscosity is relatively higher than that for the AA. This indicates the dominant influence of solution viscosity on atomization in the case of high-solid-content solutions. The impact of key operational parameters, such as AA, SR, and PA, was experimentally analyzed using the SprayWatch system in a study by [34]. The findings indicate that both a higher AA-to-SR ratio and a lower AA-to-PA ratio contribute to producing smaller droplet sizes. When optimized together, these ratios enable sprays with uniform velocity and droplet size distribution across the wetted surface. Additionally, maintaining a consistent mean droplet size is possible across various coating suspensions and spray rates [34]. The AA/PA mass flow ratio affects both the spatial distribution of droplets within the spray zone and the width of the wetted area. A higher PA results in a wider spray cone angle, leading to broader spray coverage, which may enhance coating uniformity [103].

### 5.3. Spray Drying Model

As a droplet exits the nozzle and travels toward the tablet bed, it loses some of its water content due to differences in temperature and humidity between the droplet and the surrounding drying air. The relative velocity between the droplet and the surrounding air enhances mass transfer. In the absence of such a velocity difference, mass transfer would still occur, but at a slower rate. This phenomenon, known as spray drying, is important to study as it directly affects the coating process. The spray solution consists of water and solid components, and any loss of water influences evaporation kinetics, ultimately leading to film formation occurring at a lower droplet water content [104]. As water evaporates from the droplet’s surface, its mass decreases, leading to a proportional reduction in droplet radius. The rate at which the radius decreases (Equation (26)) can subsequently be determined based on the evaporation rate (Equation (27)) [105]. The evaporation process is driven by mass transfer, as outlined earlier in Section 4.1.2. Section Balancing Complexity: Incorporating Heat Loss and Lumped Parameter Modeling. The spray drying model below represents the transition from the nozzle to the tablet bed phase and accounts for the relative velocity between the droplet and the air [106]. This velocity is used in the calculations of Reynolds number (Equation (29)), as well as the mass (Equation (31)) and heat (Equation (32)) transfer coefficients. Additionally, the evaporation of water from the droplet surface can lead to droplet blowing, which is accounted for by incorporating the Spalding number, $B_{T}$, into the heat and mass transfer coefficient equations. Another distinction from the model described earlier in Section 4.1.2 is that the surface area of a sphere with an equivalent droplet diameter is used in the evaporation rate calculation. Lastly, to account for resistance to saturated surface conditions caused by the formation of a film on the droplet surface, an activity coefficient is introduced in the calculation of the water mole fraction at the surface as presented in Equation (33).

(26) $$\frac{dr_{d}}{dt}=\frac{1\;dm_{w}}{4\;\pi \;{r_{d}}^{2}\;\rho_{w}\;dt}$$

(27) $$\frac{dm_{w}}{dt}=-k_{c}\;4\;\pi \;{r_{d}}^{2}\;\frac{M_{w}\;P_{tot}}{R\;T_{av}}\;\left(y_{vsur}-y_{v\inf}\right)$$

(28) $$C_{D}=\frac{24}{Re}+3.364\;{Re}^{-0.3471}+\frac{0.4607Re}{Re+2682.5}$$

(29) $$Re=\frac{2\;r_{d}\;\left|V_{d}-V_{a}\right|\;\rho_{g}}{\mu_{a}}$$

(30) $$B_{T}=\frac{C_{p,G}\;\left(T_{G}-T_{d}\right)}{\Delta H_{\mathrm{vap},s}}$$

(31) $$Sh=\frac{2+0.6\;{\left(Sc\right)}^{1/3}\;{\left(Re\right)}^{1/2}}{{\left(1+B_{T}\right)}^{0.7}}$$

(32) $$Nu=\frac{2+0.6\;{\left(Pr\right)}^{1/3}\;{\left(Re\right)}^{1/2}}{{\left(1+B_{T}\right)}^{0.7}}$$

(33) $$\frac{P_{s}^{\mathrm{sat}}\;a_{w}}{P_{tot}}=y_{v,sur}$$

By performing an energy balance and assuming a uniform temperature distribution within the droplet, the average temperature of the droplet can be determined by Equation (34).

(34) $$\frac{dT_{d}}{dt}=\frac{h\;4\pi {r_{d}}^{2}\;\left(T_{G}-T_{d}\right)+\Delta H_{\mathrm{vap},s}\;\frac{dm_{w}}{dt}}{m_{s}\;C_{p,s}+m_{w}\;C_{pw}}$$

Thermodynamic models typically treat droplets as a bulk mass flux entering a defined control volume, which is the tablet bed. Therefore, integrating a spray drying model, such as that of Niblett et al. [105], into a thermodynamic model could be a beneficial approach to account for droplet size distribution and determine the water content of a droplet before it reaches the tablet bed at any stage of the process.

## 6. Pan Coating Scale-Up Approaches

**Figure 6:**
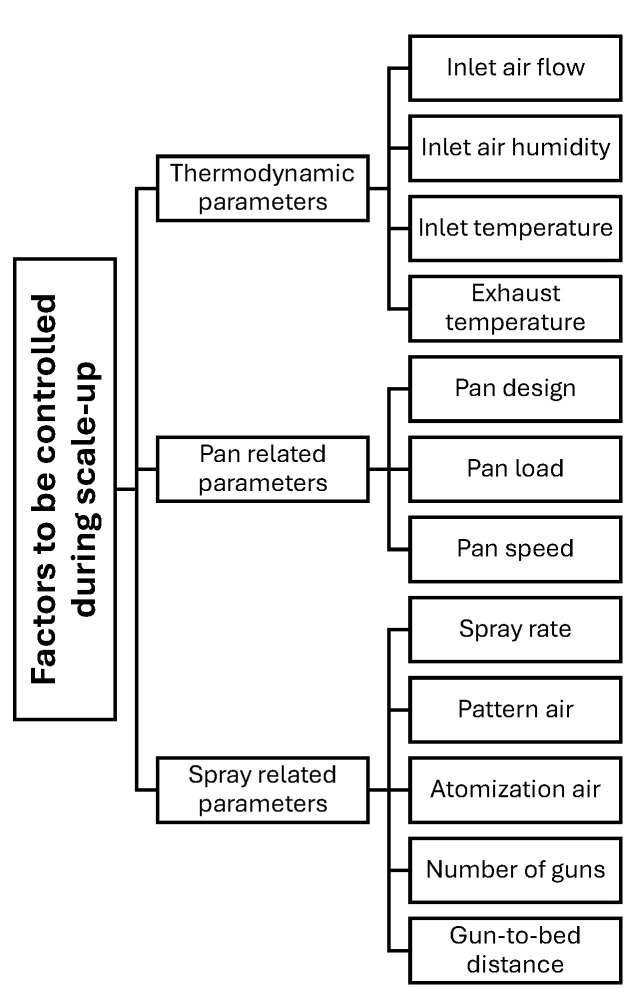
Important process-related factors to account for during the scale-up of the pan coating process.

Scaling up the pan coating process is necessary to meet industrial production requirements. However, because it involves handling numerous interdependent process variables, scaling up the process is complex [66]. The factors to consider when scaling up the pan coating process are shown in Figure 6.

Effective scale-up requires maintaining three key types of similarity: geometric, dynamic, and kinematic [50,107].

- Geometric similarity ensures that all proportional relationships between dimensions remain the same across different scales.
- Dynamic similarity involves maintaining the balance of forces governing tablet motion, such as inertial and gravitational forces.
- Kinematic similarity ensures that velocity ratios at corresponding points in the pan remain consistent across scales.

Additionally, the scale-up process can be approached from both a macroscopic and microscopic perspective [31]:

- Macroscopic approach: considers large-scale factors like heat and mass transfer, pan geometry, and spray rate to ensure consistent conditions across different scales.
- Microscopic approach: focuses on local interactions, such as how droplets interact within the spray zone and how tablets move, aiming to improve coating uniformity at a more precise level.

By combining macroscopic (bulk process parameters) and microscopic (localized interactions) approaches, an effective scale-up strategy can be achieved, ensuring consistent coating quality across different batch sizes and coater types.

### 6.1. Geometric Similarity-Pan Load

Pan load is typically defined by the volume of the tablets rather than their weight. In geometrically similar coaters, maintaining a constant ratio of pan load to pan volume (*h*/*D*) ratio) is crucial. *D* and *h* represent the pan diameter and the shortest distance from the pan’s center to the bed surface, respectively [50].

(35) PanLoad/PanVolume=constant

To preserve geometric similarity across scales, the height, width, and shape of passive baffles should remain proportionally consistent across different scales. This follows the macroscopic approach, ensuring uniform mixing and coating [31].

### 6.2. Dynamic Similarity-Pan Speed

Tablet motion within the pan is governed by inertial and gravitational forces. The Froude number (Fr) represents the ratio of these forces and can be calculated using Equation (36), where ω is the pan speed, *D* is the pan diameter, and g is gravitational acceleration [50].

(36) $$Fr=\omega^{2}\;D/g=constant$$

Maintaining a constant Fr across scales ensures that the ratio of centrifugal to inertial forces remains the same, leading to similar tablet motion patterns. This supports both macroscopic (bulk mixing) and microscopic (tablet trajectories) considerations in scaling.

### 6.3. Kinematic Similarity—Tablet Velocity and Spray Kinetics

Kinematic similarity ensures consistent velocity ratios at corresponding points in the pan across different scales. A key aspect is maintaining a constant ratio between tablet residence time on the bed surface $\tau_{\mathrm{surface}}$ and droplet drying time $\tau_{\mathrm{dry}}$, as depicted in Equation (37). This ensures that droplets dry at the same rate relative to the movement of tablets, preventing issues such as over-wetting or spray drying [107].

(37) $$\tau_{\mathrm{dry}}/\tau_{\mathrm{surface}}=constant$$

$\tau_{\mathrm{surface}}$ can be characterized based on tablet velocity and pan size. Furthermore, $\tau_{\mathrm{dry}}$ can be obtained using heat and mass transfer and setting the target tablet moisture content [105].

#### 6.3.1. Spray Dynamics

Maximizing the spray rate (SR) is desirable for process efficiency, but several factors must be maintained during scale-up, as described in Equation (38) [50].

(38) $$\left(SR\right)\times \left(\frac{n_{tablets}}{N_{t}}\right)=\mathrm{constant}$$

With larger drums, the increase in the total number of tablets is typically more significant than the increase in the number of tablets in the spray zone. This causes tablets to spend more time in the drying zone before reappearing in the spray zone. Therefore, tablets in a larger pan can be sprayed with more solution during each spraying cycle [107].

**Figure 7:**
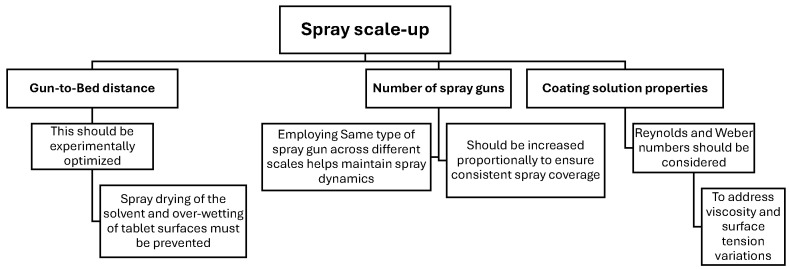
Key considerations for spray scale-up.

Following the adjustments mentioned in Figure 7 aligns with both macroscopic (spray-to-airflow balance) and microscopic (droplet dynamics) approaches [31].

#### 6.3.2. Coating Time

The duration of spraying, $T_{spraying}$, is one of the critical factors that affect tablet weight gain. A fundamental scaling relationship is shown in Equation (39).

(39) $$T_{spraying}\times SR/pan\;load=constant$$

To maintain a uniform coating weight, the average number of passes under the spray gun ($N_{c}$) should be constant. By ensuring consistent $N_{c}$, all tablets receive equal coating instances, preventing inter-tablet variability [107].

(40) $$N_{c}=\frac{V\;L\;J\;t}{a\;N_{t}}$$

## 7. Data Collection and Process Analytical Technologies

### 7.1. Data Logging to Understand Thermodynamic Micro-Environment

Temperature and humidity inside the pan play a vital role in the coating process. Conventionally, it is monitored through inlet and exhaust conditions (temperature and dew point), with sensors placed outside the coating pan. Internal bed conditions are then estimated from exhaust air data. The ability to directly monitor tablet and air temperatures and humidities inside the pan provides more detailed information, enabling a clearer understanding of the impact of key process parameters (exhaust temperature, pan speed, spray rate) on both process dynamics and the final coating quality [69].

Okutgen et al. [108], employing fixed thermocouples and humidity indicators, concluded that inlet air temperature and sensor location within the tablet bed have a significant influence on the thermodynamic conditions (temperature and humidity) of the tablet bed. Furthermore, Wobker et al. [67] used tablet-size sensors (PyroButtons^®^) moving freely within the tablet bed and compared their recordings with those of fixed sensors. The outcome was that moving sensors represented a cooler and wetter tabled bed compare with the fixed ones. Building on this, Pandey et al. [69] investigated the temperature and humidity of the tablet bed and exhaust air during the coating process in a 24-inch Thomas Engineering Compulab coater by placing PyroButton^®^ data loggers at various locations, including the exhaust plenum, spray gun bar, and baffles. Additionally, some data loggers were allowed to move freely within the tablet bed to capture dynamic variations in temperature and humidity. To assess the impact of process parameters, a full-factorial design of experiments (DOE) was conducted, focusing on pan speed, exhaust temperature, and spray rate. The study included a total of ten coating runs, incorporating one low level, one high level, and two center-point repetitions for each parameter.

A consistent observation in the literature is that the exhaust air temperature is slightly higher than the tablet bed temperature, typically by a few degrees Celsius (reported as 2–3 °C in [108] and 1–5 °C in [23], as outlined earlier in Section 4.1.2. Analysis of the data recorded by loggers indicates that the temperature difference between the exhaust air and the tablet bed is influenced by process parameters such as the spray rate.

Exhaust air temperature is widely recognized as the main parameter monitored and controlled in the coating process. Nonetheless, logger measurement data in this work indicated that controlling exhaust air temperature alone would not be sufficient to completely understand the micro-environment of the tablet bed. This is because the same exhaust air temperature can be achieved through different combinations of process parameters. Consequently, tablet bed temperature and relative humidity emerge as more critical factors influencing the coating process. The dependence of tablet bed relative humidity on exhaust air temperature becomes particularly pronounced at higher spray rates.

The significance of monitoring the tablet bed micro-environment becomes more evident when logo-bridging defects are observed when the tablet bed relative humidity exceeds a specific critical value. This threshold is formulation- and tablet-dependent. In this study, for an HPMC-based Opadry coating system, the critical relative humidity was determined to be 30%.

### 7.2. Process Analytical Technologies

Effective process control is crucial in the pharmaceutical industry to minimize coating defects, reduce batch failures, and enhance overall process performance and capability. Traditional control strategies, which rely on extensive end-product testing and strict control over material attributes and process parameters, often fall short in ensuring real-time quality assurance [109]. A more advanced approach is promoted by regulatory guidelines such as those from the International Council for Harmonisation (ICH), emphasizing systematic process understanding and risk-based quality management [110]. Within this framework, Process Analytical Technology (PAT) plays a pivotal role by enabling real-time monitoring and control of critical quality attributes (CQAs) and process parameters, facilitating a shift towards real-time release testing [111]. PAT encompasses a suite of advanced analytical tools, including spectroscopic (e.g., NIR spectroscopy, Raman spectroscopy), imaging (e.g., terahertz pulse imaging, magnetic resonance imaging), and microscopic techniques (e.g., confocal laser scanning microscopy, scanning electron microscopy), all of which provide valuable insights into the film coating process [112]. Gas chromatography (GC) and headspace gas chromatography (HS-GC) can also be used to measure residual solvents in tablets coated by the pan coating process [113,114]. By integrating PAT tools and, when needed, chromatographic methods into tablet pan coating, manufacturers can achieve enhanced control, improve product quality, and ensure regulatory compliance, ultimately transforming coating operations from reactive to predictive and proactive approaches [111,115].

PAT measurements can be categorized into in-line, on-line, at-line, and off-line techniques based on their integration with the manufacturing process. In-line measurements occur directly within the coater, providing continuous, real-time data without sample removal, making them ideal for dynamic process control. Online techniques involve automated sample extraction during the coating process, analysis, and return to the process, offering rapid feedback while minimizing interference with production. At-line methods require manual or semi-automated sample collection and analysis near the production line, balancing speed and flexibility. Offline measurements, in contrast, involve sample collection and analysis in a separate laboratory setting, typically used for detailed quality assessments that do not require immediate process adjustments. The choice of PAT mode depends on process requirements, analytical complexity, and the desired level of real-time control in the tablet pan coating process [111,112,115,116,117].

#### 7.2.1. Near-Infrared Spectroscopy

Near-infrared spectroscopy (NIRS) operates within the electromagnetic spectrum wavelength range of 780 to 2500 nm. This spectral region is associated with absorption phenomena resulting from molecular vibrations, including overtone and combination of fundamental vibrational modes associated with hydrogen bonds [118]. NIRS is the most widely employed PAT tool in the film coating process, facilitating real-time monitoring of critical quality attributes such as coat weight uniformity, active pharmaceutical ingredient (API) content, and moisture levels [1,119]. An overview of Near-infrared spectroscopy applications in the pharmaceutical coating process is summarized in Table 4.

A key advantage of NIRS lies in its ability to perform non-destructive measurements within a very short time frame, enabling the rapid generation of large datasets in real time. Furthermore, NIR spectroscopy provides comprehensive insights into multiple process parameters, including coating thickness, endpoint determination, and uniformity, making it an effective PAT tool for process monitoring. Additionally, NIRS can be utilized during the coating process to track moisture levels, allowing for real-time adjustments to mitigate potential issues such as tablet sticking or coating defects like cracking [116,120,121].

Despite its advantages, NIRS has certain limitations. Its implementation necessitates prior calibration with a reference method, a process that is both time-intensive and requires expertise in multivariate data analysis and spectral pre-processing. Moreover, its high sensitivity to moisture content, while beneficial for accurate water quantification, can interfere with the assessment of other critical quality attributes [122,123]. Moreover, in-line NIR spectroscopy is often insufficient for assessing intra- and inter-coating variability [124]. In contrast, at-line NIR analysis using a tablet holder enables the evaluation of inter-tablet coating uniformity and thickness differences between the two sides of the tablet [125].

**Table 4:** Applications of Near-infrared spectroscopy for the monitoring of film coating unit operations (grouped by CQA and reference method).

Measurement	CQAs	Reference Method	References
In-line	Real-time endpoint detection of coating process	-	[126]
At-line/In-line/Off-line/On-line	Coating thickness	Optical microscopy	[127,128,129,130,131,132,133,134,135]
NIR chemical imaging	Coating thickness Coating defects	Terahertz pulsed imaging	[136,137]
Off-line	API distribution uniformity	-	[138]
NIR chemical imaging (NIR-HSI)	API content Amount of coating in coated tablet	HPLC UV-spectroscopy	[139]
In-line	Moisture content Coating percent	Loss on drying Weight gain	[140]
In-line	Weight gain of tablet	micro-CT (correlated with coating thickness)	[141]
Off-line	Color uniformity Coating uniformity Real-time endpoint detection of coating process	Optical microscopy Weight gain	[135]
On-line	Moisture absorption rate Coating Weight gain	Gravimetric analysis Weight gain	[142]
At-line/Off-line	Drug release rate	Dissolution test	[128,131,132]

#### 7.2.2. Raman Spectroscopy

Raman spectroscopy quantifies the intensity of light scattered as a function of the difference in wavenumber between the incident and scattered radiation. This shift in the wavenumber arises from the vibrational energy transitions of molecules [118]. Raman spectroscopy serves as a complementary technique to Near-Infrared Spectroscopy (NIRS) and enables the analysis of solid samples in the presence of water, as water exhibits weak Raman scattering properties [143].

**Figure 8:**
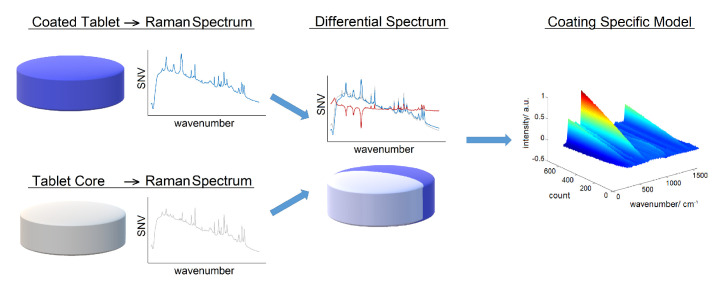
Simplified representation of model development for Raman spectroscopy [Reproduced from Radtke, Juliana, et al., Pharmaceutics; published by MDPI, 2020 [144]].

Raman and NIR spectra exhibit similarities, and for both qualitative and quantitative analysis, identical data pretreatment techniques, as well as univariate or multivariate data analysis methods (such as Principal Component Analysis (PCA) and Partial Least Squares (PLS)), can be applied, as schematically depicted in Figure 8 [118,124,144]. Furthermore, Raman spectroscopy may offer better capability for analyzing APIs compared to NIR, as many active compounds possess aromatic or conjugated structures, which exhibit strong Raman scattering [143]. An overview of Raman spectroscopy applications in the pharmaceutical coating process is summarized in Table 5.

One key challenge in Raman spectroscopy is the dominance of Rayleigh scattering, where most of the incident light is scattered elastically, while only a small fraction undergoes inelastic scattering. Although Rayleigh scattering can be minimized using interference filters or spectrometers, the issue of weak Raman scattering persists. To enhance the signal, increasing the energy of the incident light either through higher laser intensity or shorter wavelengths can be employed. However, these approaches may lead to increased sample heating or even material decomposition. Additionally, the use of lasers with shorter wavelengths may induce fluorescence, which can mask the Raman signal [143,145]. Similar to near-infrared (NIR) spectroscopy, Raman spectra must be calibrated with a reference method before being used in process monitoring [116].

Kim et al. [146] employed Raman imaging analysis to monitor the formation of coating layers during the coating process. They explored the feasibility of using in-line Raman spectroscopy as an alternative to traditional balance weighing for determining the endpoint of the coating process. To assess the accuracy of both methods, they performed *t*-tests and F-tests on two sample groups. The statistical results revealed no significant difference between in-line Raman spectroscopy and conventional balance weighing in terms of accuracy [146].

In recent years, Raman spectroscopy has been successfully implemented as a PAT tool for active coatings [47,147], colored coatings [148], and multilayered film coatings on pellets [149].

**Table 5:** Applications of Raman spectroscopy for the monitoring of film coating unit operations (grouped by CQA and reference method).

Mode of Operation	CQAs	Reference Method	References
At-line	Coating thickness	Coating time	[150]
		Digital micrometer	[151]
		Weight gain	[152]
		Optical microscopy	[129]
In-line	Coating thickness	Terahertz pulsed imaging	[153]
		Geometric model calculation	[148,154]
		Weight gain	[146]
In-line	Drug release	Dissolution test	[153]
In-/off-line	Coating thickness Drug content	Optical microscopy HPLC	[155]
On-line	Coating thickness	Optical microscopy	[156]

#### 7.2.3. Terahertz Pulsed Imaging

Terahertz Pulsed Imaging (TPI) and terahertz spectroscopy operate within the far-infrared region of the electromagnetic spectrum (0.06–3 THz), bridging microwaves and infrared radiation [157]. TPI offers the advantage of being a non-destructive technique and enables film thickness measurement without the need for a chemometric calibration model [118]. These techniques rely on the generation and detection of terahertz pulses, which interact with the tablet coating, enabling the measurement of time-of-flight and amplitude changes. Through analysis of the terahertz temporal waveform in the time domain, coating thickness (*d*) can be non-destructively determined using the time delay (Δt) between pulse reflections at the coating surface and the coating–core interface [158]. This calculation, as shown in Equation (41), incorporates the refractive index of the coating material ($n_{refract}$) and the speed of light (*c*), allowing precise characterization of coating properties. The refractive index can be determined through terahertz spectroscopy in transmission mode [159].

(41) $$d=\frac{\Delta t\;c}{2\;n_{refract}}$$

TPI measurements can be performed at a single point within the investigated area, requiring only a few milliseconds for acquisition [124], or across multiple locations to map the entire surface of the tablet. The main disadvantage of full scan mode is the long acquisition time, which typically ranges from 20 to 50 min [160] and can take up to 60 min [161].

TPI and terahertz spectroscopy applications in the pharmaceutical coating process are summarized in Table 6.

#### 7.2.4. Image Analysis

Spectroscopic methods, such as NIRS, Raman spectroscopy, and terahertz pulsed imaging, commonly used to measure tablet coating thickness, can primarily provide average values without capturing coating distribution on individual tablets. Advances in hyperspectral imaging now enable nearly complete surface analysis, though they generate large datasets that require sophisticated processing. The use of multivariate image analysis (MIA) with a webcam inside a coating pan to assess coating uniformity was demonstrated by [172]. However, frequent equipment disassembly in pharmaceutical manufacturing necessitates model recalibration for each batch. Furthermore, the increasing complexity of imaging data has led to the adoption of machine learning techniques, such as support vector machines (SVM) and convolutional neural networks (CNN), to enhance data processing [173,174]. Additionally, advancements in imaging and lighting technologies have led to the development of smaller, more durable, and cost-effective imaging systems capable of capturing high-resolution images, enhancing the feasibility of real-time coating analysis.

The near-infrared chemical imaging (NIR−CI) technique has been recognized as a reliable and effective tool for quality control in pharmaceutical development and manufacturing [175,176,177,178,179]. NIR−CI is derived from conventional NIR spectroscopy and offers the advantage of capturing a substantial amount of both spectral and spatial data within a single image. However, since the measurement process requires a few minutes, its application is limited to at-line or off-line analysis rather than real-time monitoring [139]. Moreover, studies by Palou, Anna, et al. [180] and Kandpal et al. [181] have demonstrated its capability in evaluating coating and API uniformity, respectively [180,181]. Rodrigues et al. [182] evaluated the effectiveness of a single covariance-based MIA model combined with a stability determination strategy for in-line endpoint detection in the film coating of colored tablets. When applied to high-quality images, the algorithm’s results closely aligned with those from visual inspection, with minimal errors. While the MIA model demonstrated robustness against variations in processing conditions, reducing the need for frequent recalibration, adjustments would still be necessary in cases of substantial changes to tablet cores, coating solutions, imaging equipment, lighting conditions, or coating machinery [182].

#### 7.2.5. Optical Coherence Tomography

Optical Coherence Tomography (OCT) is a technique capable of generating cross-sectional, depth-resolved images in either two or three dimensions for examined samples [183,184]. This non-destructive, high-resolution imaging method is particularly suitable for assessing and monitoring pharmaceutical film coatings.

OCT facilitates real-time acquisition of detailed surface images of tablet coating layers, making it a valuable tool for in-line assessment of intra- and inter-tablet coating uniformity [184]. Additionally, this technique has gained significant interest in pharmaceutical manufacturing since this technique does not require chemometric model calibration before measurements [124].

However, one limitation of OCT is its inability to penetrate thick coatings exceeding 200 μm or to reveal internal tablet structures [185]. Most applications focus on single-layer coatings, typically ranging from 50 to 100 μm in thickness. Recent research suggests that integrating OCT with NIRS and TPI can help overcome these limitations, enabling a more comprehensive analysis of the coating layer [185,186,187].

**Figure 9:**
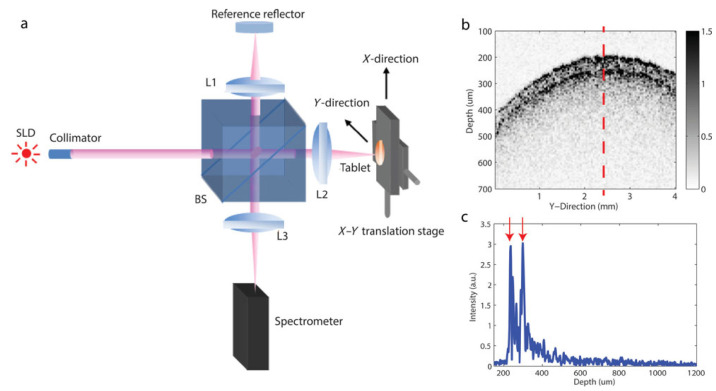
Schematic of a custom SD-OCT system with an X-Y translation stage for tablet positioning (**a**). The system captures 100 × 100 B-scans (**b**), where each B-scan is a cross-sectional image formed by combining multiple A-scans, which are depth profiles showing reflection intensity at different depths. An example scan highlights the tablet’s central region, with the red dashed line indicating the lateral position selected for A-scan extraction and arrows denoting the corresponding interfaces in the extracted A-scan (**c**). [Reproduced from Lin, Hungyen, et al., Journal of Pharmaceutical Sciences; published by Wiley Periodicals, Inc. and the American Pharmacists Association, 2015 [188]].

Developments in OCT have demonstrated its applicability in monitoring film coatings during the pan coating process. Spectral-domain OCT (SD-OCT) systems (Figure 9), known for their high spatial resolution, have been successfully implemented for both off-line and in-line measurements, enhancing their utility in pharmaceutical manufacturing [188,189]. However, the shift toward multi-particulate dosage forms has heightened the need for precise ultra-thin (below 20 μm) coating analysis, pushing the limits of conventional OCT resolution. To address these limitations, ultrahigh-resolution OCT (UHR-OCT) has emerged as a powerful technique, which is capable of capturing detailed morphological features and detecting coating defects in thin film layers. This advanced imaging approach enables precise measurement of film thickness and provides quantitative insights into coating variability, making it particularly valuable for the analysis of agglomerated pharmaceutical pellets and other modern dosage forms [190].

## 8. Summary and Future Perspectives

The tablet film coating process can be impacted by three different phenomena: tablet mixing, coating spraying, and coating evaporation (drying). Each can be studied using specific models, such as particle dynamics, spray dynamics, and thermodynamics. There has been progress, to some extent, in the advancement of sub-models specific to the individual components of the process. However, a predictive model that treats the coating process in its entirety is still missing. Such a model should be capable of capturing interactions between key mechanisms occurring during the coating process. In our opinion, the most promising improvement to the field can be made by developing an integrated tablet film coating model that is able to predict the optimum process conditions and consequently result in a smooth and uniform coating with minimal variation in inter-tablet and intra-tablet thickness.

In our opinion, prospective research should try to address the following key questions:

- How can existing sub-models (particle dynamics, spray dynamics, thermodynamics) be integrated into a unified, predictive coating model?
- What methodologies should be used in model development, calibration, and validation to ensure applicability across different coater types and scales?
- How can machine learning and data-driven approaches be developed for analyzing historical data? This can reveal the interplay between process parameters and product quality, support decision making for future coating processes, and lead toward predictive modeling
- What role can advanced PAT tools play in enabling in-line and real-time monitoring, as well as in better capturing inter-tablet and intra-tablet coating variability?
- How can mechanistic modeling and PAT-based approaches be extended to facilitate understanding of the correlations between process parameters and critical quality attributes, CQAs?

Answering these questions will be essential for bridging the current knowledge gap and moving toward robust, predictive, and quality-driven modeling of tablet film coating processes.

## Figures and Tables

**Table 1:** A grouped list of inputs for thermodynamic models.

Tablet Core Properties	Spraying System Parameters	Inlet Air Conditions	Pan Coater Characteristics
Tablet size (diameter, thickness). Batch size. Density and moisture content of the tablet.	Spray rate. Temperature of the spray solution.	Temperature. Mass flow rate. Airflow rate.	Diameter. Length. Rotational speed. Baffle geometry. Residence time of tablets in the spray zone.

**Table 2:** A review of existing thermodynamic models for tablet film coating, along with their assumptions, applications, and advancements.

References	Model Assumptions	Model Output	Model Application	Development & Advancements
am Ende et al. [54]	Tablet temperature is assumed to be the same as the exhaust air temperature.	Exhaust air temperature & humidity.	Both aqueous and organic film coating. Bi-conical coaters such as Vector LDCS-20, etc.	Early model integrating experimentally obtained HLF into the model.
Page et al. [56]	Tablet bed divided into spray and dry zones.	Exhaust air and tablet bed temperatures and humidities.	Only aqueous film coating. Cylindrical coaters such as Bohle Lab-Coater.	First model to incorporate zonal division in the bed but does not account for heat loss to the environment.
Strong [35]	Evaporative mass transfer occurs at wet-bulb temperature.	Environmental equivalency (EE) & tablet drying rate.	Steady-state operation of the coater. Theoretical (no experimental results)	First attempt to build a theoretical framework to analyze drying efficiency thermodynamically.
Prpich et al. [66]	Same as am Ende et al. 2005 [54].	Same as am Ende et al. 2005 [54].	Only aqueous film coating. Bi-conical coaters such as Glatt GC 1250, etc.	Adaptation of am Ende & Berchielli (2005) [54] model to different coater types.
Rodrigues et al. [55]	Uses lumped parameters in heat and mass transfer.	Exhaust air and tablet bed temperatures and humidities.	Only aqueous film coating. Bi-conical coaters such as Accela-Cota coater.	Builds on Page et al. (2006a,b) [56] by incorporating heat loss and lumped parameter modeling while excluding zoning complexity and tablet exchange rate estimation.

**Table 3:** Strengths and limitations of introduced modeling approaches for pan coating process.

Technique	Information	Strengths/Advantages	Limitations/Disadvantages
Thermodynamic Modeling	Simulates coating process through coupled mass and energy balances between air, spray, and tablet bed.	• Facilitates virtual testing, reducing costly and time-consuming trial-and-error experiments. • Applicable across scales (lab to production), supporting process design and scale-up. • Enables optimization of critical variables (inlet air temperature, spray rate, pan speed).	• Limited representation of particle-scale dynamics (mixing, residence time distribution). • Spray-related factors (nozzle number, angles, spray zone coverage, pattern air) not included [54]. • Challenges in modeling droplet size and wetting behavior, accurate modeling of droplet size and wetting requires specialized measurements [34]. • Cannot capture real-time process variability (e.g., nozzle clogging, air fluctuations). • Simplified assumptions for heat and mass transfer, ignoring local variations in the bed. • Mechanical defect mechanisms (twinning, orange peel, overwetting) excluded.
Discrete Element Modeling (DEM)	Simulates tablets movement and mixing using Newton’s equations of motion.	• Captures detailed particle motion, mixing, and residence time distribution. • Provides insights into intra- and inter-tablet coating variability. • Can model non-spherical particles with glued sphere approach [89]. • Effect of different tablet shapes and drum geometry (e.g baffle shape and number, spray zone coverage) can be investigated by the DEM [27].	• Computationally very expensive, especially for industrial-scale coaters [90]. • Requires calibration of input parameters (friction, restitution, cohesion), which are difficult to measure. • Coupling with a postprocessing approach (e.g., PBM, DDM, MC, ray-tracing) is needed to obtain particle-level information [74].
Population Balance Models (PBM)	Considers coating formation as the accumulative result of repetitive random passes through the spray zone.	• Provides information on coating mass distribution among tablets as a function of time (inter-tablet variability). • Effective for examining how changes in process parameters affect qualitative trends [90]. • Computationally efficient compared to DEM.	• Dependent on experiments or other simulation methods to determine a priori parameters β and $Q_{c}$. • PBM is based on compartmental and exchange models, which might not fully capture the complex 3D dynamics of the tablets, spray dynamics or detailed droplet behavior [90]. • PBM alone is incapable of capturing intra-tablet uniformity. • Predictions outside the calibrated range are unreliable [90].

**Table 6:** Applications of Terahertz spectroscopy for the monitoring of film coating unit operations (grouped by CQA and reference method). Rows marked with * correspond to in-line measurements; all other rows are off-line measurements.

CQAs	Reference Method	References
Single point thickness comparison	Optical microscopy Near-infrared spectroscopy	[162]
Multipoint comparison Coating uniformity Limit of detection	Optical microscopy Near-infrared spectroscopy	[127]
Intra-tablet variation in coating thickness	-	[160]
Coating uniformity and morphology	Dissolution test	[163]
Coating thickness distribution and defects	NIR chemical imaging	[136]
Intra-batch coating thickness distribution *	Off-line terahertz imaging Weight gain	[161]
Coating layer density Coating/core interface	-	[164]
Coating thickness and density	Dissolution test Weight gain	[165,166]
Coating morphology and defects	SEM	[167]
Coating thickness and uniformity Coating interface and morphology	X-ray microtomography	[168]
Coating thickness *	Off-line terahertz spectroscopy	[169]
Coating layer thickness Coating interface and morphology	X-ray microtomography	[170]
Dissolution of immediate release film coating	-	[171]

## Data Availability

Not applicable.

## Glossary

*AA*	Atomization air flow rate
$A_{P}$	Circular area of inner ribbon covered by tablets (m^2^)
$A_{\mathrm{film}}$	Surface area of the film (m^2^)
$A_{drum}$	Surface area of the drum through which heat is lost (m^2^)
*A*	Surface area over which heat transfer occurs (m^2^)
$B_{T}$	Spalding number
*CFD*	Computational fluid dynamics
*CQAs*	Critical quality attributes
*CV*	Coefficient of variation
$C_{D}$	Drag coefficient of the droplet
$C_{G}$	Concentration of solvent in the gas phase (kg·m^−3^)
$C_{p,s}$	Specific heat capacity of solvent in coating solution (J·kg^−1^ · K^−1^)
$C_{p,w}$	Specific heat capacity of water (J/kg·K)
$C_{w}$	Concentration of solvent in the film interface (kg·m ^−3^)
$C_{1}$	Empirical constant in the atomization correlation
$C_{2}$	Empirical constant in the atomization correlation
$C_{p,G}$	Specific heat capacity of gas (=air) (J/kg·K)
*DDM*	Discrete drop method
*DEM*	Discrete element method modeling
$D_{l}$	Diameter of the liquid nozzle
*D*	Pan diameter (m)
*EE*	Environmental equivalency
$F_{r}$	Froude number
*G*	Growth rate of coating mass
*HLF*	Overall heat loss from the coater (W)
*H*	Drum length (m)
*J*	Number of spray guns
*L*	Length of the spray zone (m)
*MC*	Monte Carlo approach
$M_{S}$	Molar mass of the solvent (kg·mol ^−1^)
$M_{w}$	Molar mass of water (kg/mol)
$M_{tp1}$	Mass of tablets in zone 1 (kg)
$M_{tp2}$	Mass of tablets in zone 2 (kg)
NIR−CI	Near-infrared chemical imaging
NIRS	Near-infrared spectroscopy
$N_{c}$	Average number of passes of a tablet under the spray gun
$N_{i}^{1}$	The total number of particles in class $C_{i}$ at time *t* in the spray zone
$N_{i}^{K}$	The total number of particles in class $C_{i}$ at time *t* in mixer *k*
$N_{t}$	Total number of tablets in the bed
$N_{i-1}^{N}$	The total number of particles in class $C_{i-1}$ at time *t* in mixer *N*
$N_{u}$	Nusselt number of the droplet
*N*	Total number of compartments
*OCT*	Optical Coherence Tomography
*Oh*	Ohnesorge number of the liquid stream
*PAT*	Process analytical technology
*PA*	Pattern air flow rate
*PBM*	Population balance modeling
$P_{s}^{\mathrm{sat}}$	Saturated vapor pressure of the solvent (Pa)
$P_{tot}$	Total pressure (Pa)
$P_{r}$	Prandtl number of air
$Q_{c}$	Rate of particle exchange between perfect mixers
$Q_{l}$	Spray rate (kg/s)
$Q_{HLF}$	Overall heat loss from the coater (W)
*RSD*	Relative standard deviation
$Re_{b_{g}}$	Reynolds number of the air stream (based on outlet width $b_{g}$)
Re	Reynolds number of the droplet
*R*	Universal gas constant (J·K^−1^·mol^−1^)
*SMD*	Sauter mean diameter of droplets
*SR*	Spray rate (kg/s)
*Sc*	Schmidt number
*Sh*	Sherwood number of the droplet
*TPI*	Terahertz Pulsed Imaging
$T_{G}^{*}$	Effective temperature of the gas phase (K)
*TG*	Temperature of the gas phase (K)
$T_{\mathrm{coat}}$	Coating solution temperature (K)
$T_{w}$	Temperature at the film interface (K)
$T_{D}$	Drum temperature (K)
$T_{G,in}$	Inlet gas temperature (K)
$T_{G,out}$	Outlet gas temperature (K)
$T_{Surr}$	Room (surrounding) temperature where the coater is located (K)
$T_{av}$	Average temperature of the droplet (K)
$T_{d}$	Temperature of the droplet (K)
$T_{spraying}$	Spraying duration (s)
$T_{tb}$	Temperature at the tablet surface (K)
*T*	Temperature (K)
$U_{L\to G}$	Lumped mass transfer coefficient, product of evaporative
	surface area and mass transfer coefficient (kg·s^−1^)
*U*	Overall heat transfer coefficient (W·m^−2^·K^−1^)
$V_{P}$	Volume of tablets between ribbons (m^3^)
$V_{a}$	Air velocity (m/s)
$V_{d}$	Droplet velocity (m/s)
*V*	Velocity of the tablet in the spray zone (m/s)
$We_{D_{l}}$	Weber number of the liquid stream (based on nozzle diameter $D_{l}$)
$X_{s}$	Concentration of coating solution (kg/kg)
$x_{s}$	Solvent fraction in the coating solution
$\Delta H_{\mathrm{vap},s}$	Latent heat of vaporization of the solvent (J/kg)
Δt	Time delay between pulse reflections at the coating surface and coating–core interface (s)
$\alpha_{G\to tb}$	Convective heat transfer coefficient between gas and tablet bed (W·m^−2^·K^−1^)
$\alpha_{L,(G\to tb)}$	Lumped convective heat transfer coefficient between gas and tablet bed (W·m^−2^·K^−1^)
β	Fraction of particles present in the cascading zone
${\dot{m}}_{\mathrm{coat}}$	Mass flow rate of coating solution (kg/s)
${\dot{M}}_{evap}$	Mass flux between the film and the gas phase (kg·s^−1^)
${\dot{m}}_{G,in}$	Inlet gas mass flow rate (kg/s)
${\dot{q}}_{G\to tb}$	Heat flux from the gas phase to the tablet bed (W)
CSTR	Continuous stirred-tank reactor
RH	Relative humidity of the drying air
$\mu_{a}$	Dynamic viscosity of air (Pa·s)
$\phi_{S}^{surf}$	Surface fraction of solvent in the film
$\psi_{1}\left(x,t\right)$	Population density of tablets in the spray zone with coating mass between *x* and x+dx at time *t*
$\psi_{2}\left(x,t\right)$	Population density of tablets in the drying zone with coating mass between *x* and x+dx at time *t*
$\psi_{e}^{1}$	Population density at the exit of spray zone
$\psi_{e}^{k}$	Population density at the exit of mixer k of dry zone
$\psi_{i}^{1}$	Population density at spray zone
$\psi_{i}^{k}$	Population density at mixer k of dry zone
$\psi_{j}$	Exchange rate of tablets between zones
$\psi_{s}^{1}$	Population density at the start of spray zone
$\psi_{s}^{k}$	Population density at the start of mixer k of dry zone
$\rho_{w}$	Density of water (kg/m^3^)
$\rho_{bulk}$	Bulk density of tablets (kg/m^3^)
$\rho_{g}$	Density of the gas (air) stream
$\rho_{l}$	Density of the liquid stream
$\tau_{\mathrm{dry}}$	Droplet drying time (s)
$\tau_{\mathrm{surface}}$	Tablet residence time on the bed surface (s)
τ	Time (s)
$a_{w}$	Water activity of the droplet surface
*a*	Projected area of a tablet (m^2^)
$b_{g}$	Width of the atomizing air outlet
$c_{\psi_{j}}$	Exchange rate constant, defining the fraction of volume $V_{P}$ exchanged per drum rotation
*c*	Speed of light (m/s)
*d*	Coating thickness (m)
$h_{\mathrm{cond}}$	Convective heat transfer coefficient (W/m^2^·K)
*h*	shortest distance from the pan’s center to the bed surface (m)
$k_{c}$	Mass transfer coefficient (m·s^−1^)
$m_{D}^{*}$	Effective drum mass (kg)
$m_{s,\mathrm{drop}}$	Mass of solvent in the droplet (kg)
$m_{w}$	Mass of water in the droplet (kg)
$m_{s}$	Mass of the solvent in the film (kg)
$m_{r}$	Mass flux ratio between fluid streams
$n_{i}^{1}$	Fraction of particles in class $C_{i}$ at time *t* in the spray zone
$n_{i}^{N}$	Fraction of particles in class $C_{i}$ at time *t* in mixer *N*
$n_{i}^{k}$	Fraction of particles in class $C_{i}$ at time *t* in mixer *k*
$n_{i}^{k-1}$	Fraction of particles in class $C_{i}$ at time *t* in mixer k−1
$n_{s}$	Pan rotation speed (rev/s or 1/s)
$n_{refract}$	Refractive index of the coating material
$n_{tablets}$	Number of tablets in the spray zone
$r_{d}$	Droplet radius (m)
*t*	Total coating duration (s)
$y_{v,\infty }$	Vapor mass fraction in the bulk gas
$y_{v,sur}$	Vapor mass fraction at the droplet surface
